# Phage bioinformatics tools: a review of computational approaches for bacteriophage research

**DOI:** 10.1093/bib/bbag494

**Published:** 2026-09-15

**Authors:** Sean Jia Le Pang, Soon Keong Wee, Eric Peng Huat Yap

**Affiliations:** Institute for Digital Molecular Analytics and Science, Interdisciplinary Graduate Programme, Nanyang Technological University, 61 Nanyang Drive, Academic Block North, ABN-01b-11, 637335, Singapore; Institute for Digital Molecular Analytics and Science, Nanyang Technological University, 59 Nanyang Drive, Experimental Medicine Building EMB-06-35 (Level 6), 636921, Singapore; Institute for Digital Molecular Analytics and Science, Nanyang Technological University, 59 Nanyang Drive, Experimental Medicine Building EMB-06-35 (Level 6), 636921, Singapore; Lee Kong Chian School of Medicine, Nanyang Technological University, 11 Mandalay Rd, 308232, Singapore; National Centre for Infectious Diseases, 16 Jalan Tan Tock Seng, 308442, Singapore

**Keywords:** bacteriophage, bioinformatics, foundation models, protein structure, host prediction, viral metagenomics

## Abstract

Rising clinical interest in phage therapy and the exponential growth of metagenomic sequence catalogues have driven a rapid expansion of bacteriophage bioinformatics. More than 80 dedicated tools, mostly published since 2020, now span identification, assembly, annotation, taxonomy, lifestyle prediction, defence-system detection, and host prediction. Aimed at experienced practitioners and developers, this review synthesizes the field through the lens of three successive computational paradigms: sequence homology, bounded by database completeness; machine learning, constrained by labelled training data; and foundation models, which now achieve Matthews correlation coefficients above 0.95 in identification tasks and, through structure-informed prediction, raise functional annotation to over half of phage genes. Furthermore, we map the upstream components, namely, gene callers, homology engines, protein language models, and structural search tools, that underpin most downstream pipelines, exposing shared infrastructure and ecosystem-level fragility when dependencies change. To translate this into practice, we propose web-based and command-line reference workflows calibrated to user expertise and sample types. Finally, we set an agenda for the next wave of tool development. Roughly half of phage genes still resist functional annotation despite structural methods; no broadly generalizable strain-level host predictor exists for phage therapy; varying true-positive rates (0%–97%) underscore the absence of standardized community benchmarks analogous to Critical Assessment of Structure Prediction or Critical Assessment of Metagenome Interpretation. As generative genome models begin designing synthetic phages, progress will depend less on producing standalone tools than on rigorous evaluation, interoperable infrastructure, and clinically meaningful prediction targets.

## Introduction

Rising antimicrobial resistance has renewed clinical interest in bacteriophage therapy, while high-throughput sequencing has expanded phage diversity catalogues. IMG/VR has grown from ~268 000 sequences in its 2017 release to >15 million virus genomes and fragments in version 4 [1], a trajectory mirrored by curated resources such as INfrastructure for a PHAge REference database (INPHARED) [2]. This expansion supplies the training data on which modern computational tools depend. More than 80 dedicated phage bioinformatics tools now span eight functional categories: identification, assembly, annotation, taxonomy, lifestyle prediction, defence system detection, host prediction, and benchmarking, with the majority published between 2020 and 2025.

**Figure 1:**
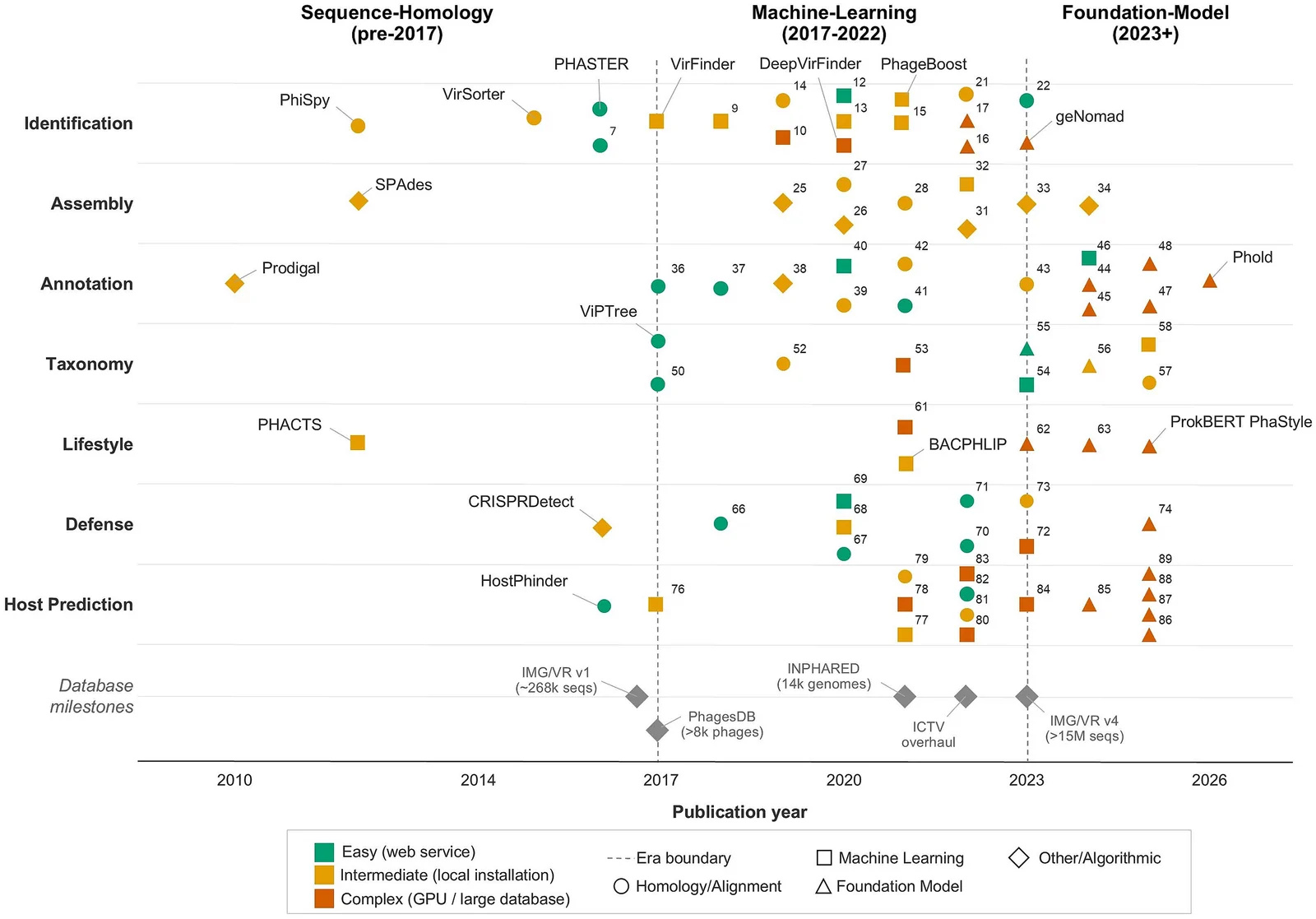
Landscape of computational methods for phage analysis across major functional tasks from 2010 till today. Timeline showing the development of tools for phage identification, assembly, annotation, taxonomy, lifestyle prediction, defence detection, and host prediction, stratified by methodological paradigm: sequence homology (pre-2017), machine learning (2017–22), and foundation models (2023+). Each point represents an individual tool positioned by publication year, with marker shape indicating algorithmic class (homology/alignment, machine learning, or foundation model) and colour denoting implementation complexity (web-based, local installation, or GPU/large-scale requirements). Dashed vertical lines mark the two era boundaries (2017 and 2023) that separate the three paradigms, each transition driven by a distinct methodological breakthrough: profile and alignment-based homology search in the first era, feature-engineered and deep-learning classifiers in the second, and protein language models with structure prediction in the third. Key database milestones are indicated along the bottom (IMG/VR v1, PhagesDB, INPHARED, the ICTV taxonomy overhaul, and IMG/VR v4), showing how successive expansions of sequence and taxonomic resources drove each new wave of tool development and underscoring the co-evolution of data resources and analytical methods.

Tool development has followed three computational paradigms (Fig. 1). The homology era (pre-2017) relied on BLAST alignments, hidden Markov model (HMM) profiles, and curated reference databases; tools such as VirSorter [3] and vConTACT2 [4] scanned sequences against known viral markers and built gene-sharing networks from shared protein clusters but lost sensitivity on short metagenomic contigs and novel lineages lacking close database homologs. The machine-learning era (2017–22) replaced explicit homology with statistical patterns learned from sequence composition; VirFinder [5] and PhaGCN [6] demonstrated reference-free identification and graph-based taxonomic classification but remained bounded by labelled training-data availability and depended on short fragments. Foundation models, defined as transformer-based models pretrained by self-supervised learning on massive unlabelled sequence corpora and then fine-tuned for specific downstream tasks, underpin the third era. Since 2023, these models, including protein and genomic language models, have pushed performance ceilings higher. With geNomad [7] performing reference-independent identification and Phold [8] introducing structure-informed functional annotation, the dark matter gap of viral genes with unknown function that defines this period is starting to close. These boundaries, however, are gradational rather than discrete, and several tools blend paradigms. Tool such as geNomad couples a neural-network sequence encoder with classical marker-protein profiles, and PhaTYP combines transformer pretraining with fine-tuning on a modest phage-specific corpus, straddling the machine-learning and foundation-model eras. We therefore assign each tool to the paradigm that best reflects its core methodology while treating the era boundaries as continuous.

Recent guides such as Phage quest [9] have introduced phage bioinformatics to biological and medical researchers new to the field. This review takes a complementary but distinct position: it is aimed at experienced users applying tools in practice and at developers building the next generation of tools. We systematically survey >80 tools organized by computational paradigm (Table 1), synthesize benchmarking evidence and identify where foundation models are beginning to address persistent gaps (ranging from viral dark matter to strain-level host prediction) for phage therapy, and identify the key limitations of current approaches, and outline future directions to guide the next phase of tool development.

**Table 1:** Bioinformatics tools for bacteriophage research.

Databases							
**No.**	**Tool**	**Year**	**Description**	**Key feature**	**Citations** **(Apr 2026)**	**Availability**	**URL**
**T1**	NCBI Viral Genomes Resource	2015	Curated complete viral genomes; RefSeq records	INSDC-linked reference standard	777	Web	https://www.ncbi.nlm.nih.gov/genome/viruses/
**T2**	PhagesDB	2017	Actinobacteriophage database (SEA-PHAGES)	>30 000 entries; >5000 sequenced genomes as of 2026	540	Web	https://phagesdb.org/
**T3**	INPHARED	2021	Automated curation of complete phage genomes	GitHub-distributed; automated updates; revealed 75% sampling bias	371	GitHub	https://github.com/RyanCook94/inphared
**T4**	ICTV VMR	2022	Exemplar virus taxonomy reference	Official nomenclature alignment	1504	Web	https://ictv.global/vmr
**T5**	IMG/VR v4	2023	Uncultivated virus genome repository	>15 million genomes; six-fold increase over v3	446	Web	https://img.jgi.doe.gov/vr/
Identification and detection							
**No.**	**Tool**	**Year**	**Approach**	**Key metric/feature**	**Citations** **(Apr 2026)**	**Availability**	**URL**
**T6**	VirSorter	2015	Hallmark gene search + enrichment metrics	>95% recall on contigs ≥10 kb	1182	GitHub	https://github.com/simroux/VirSorter
**T7**	MetaPhinder	2016	BLAST integration across reference genomes	Sequence-level classification	84	GitHub + Web	https://github.com/vanessajurtz/MetaPhinder
**T8**	VirFinder	2017	Logistic regression on *k*-mer frequencies	78× higher TPR than VirSorter at 1 kb	701	GitHub	https://github.com/jessieren/VirFinder
**T9**	MARVEL	2018	Random Forest on genomic features	Extended RF-based detection	191	GitHub	https://github.com/LaboratorioBioinformatica/MARVEL
**T10**	PPR-Meta	2019	Deep learning	Three-way classification (phage/plasmid/chromosome)	200	GitHub	https://github.com/zhenchengfang/PPR-Meta
**T11**	DeepVirFinder	2020	Alignment-free CNN classifier	AUROC 0.93–0.98 for 300–3000 bp	691	GitHub	https://github.com/jessieren/DeepVirFinder
**T12**	Seeker	2020	LSTM on raw DNA	Alignment-free; no feature engineering	137	GitHub	https://github.com/gussow/seeker
**T13**	VIBRANT	2020	Neural network + protein annotation	Automated recovery, annotation, curation	1128	GitHub	https://github.com/AnantharamanLab/VIBRANT
**T14**	Kraken2	2019	*k*-mer exact matching	Precision 0.96 in mock community (Ho *et al*. benchmark)	7218	GitHub	https://github.com/DerrickWood/kraken2
**T15**	VirSorter2	2021	Multi-classifier framework	F1 > 0.8 DNA and RNA virus detection	1279	GitHub	https://github.com/jiarong/VirSorter2
**T16**	PhaMer	2022	Transformer on protein tokens	27% F1 improvement on real metagenomic data	65	GitHub + Web	https://github.com/KennthShang/PhaMer
**T17**	INHERIT	2022	DNABERT-style transformer	Representation learning for phage genomes	29	GitHub	https://github.com/Celestial-Bai/INHERIT
**T18**	geNomad	2023	IGLOO encoder + CRF for proviruses	**MCC 95.3%; virus ID + taxonomy + annotation in one framework**	884	GitHub	https://github.com/apcamargo/genomad
Prophage detection							
**No.**	**Tool**	**Year**	**Approach**	**Key metric/feature**	**Citations** **(Apr 2026)**	**Availability**	**URL**
**T19**	PhiSpy	2012	Similarity + composition (7 genomic features)	94% prediction success; 0.66% FPR across 50 genomes	593	GitHub + Web	https://github.com/linsalrob/PhiSpy
**T20**	PHASTER	2016	Curated database search (web server)	Widely used prophage web server	3855	Web	https://phaster.ca/
**T21**	DEPhT	2022	Multimodal approach (3 run modes)	Improved prophage boundary determination	38	GitHub	https://github.com/chg60/DEPhT
**T22**	PHASTEST	2023	Updated PHASTER pipeline	31% faster; higher sensitivity than PHASTER	554	Web	https://phastest.ca/
**T23**	PhageBoost	2021	Machine-learning-driven (evaluates viral genomic architecture)	Detects highly divergent prophages; fast, for high-throughput WGS	74	GitHub	https://github.com/ku-cbd/PhageBoost
Genome assembly and comparative genomics							
**No.**	**Tool**	**Year**	**Approach**	**Key metric/feature**	**Citations** **(Apr 2026)**	**Availability**	**URL**
**T24**	SPAdes	2012	de Bruijn graph assembly	Foundation for metaSPAdes/metaviralSPAdes	27 729	GitHub + Web	https://github.com/ablab/spades
**T25**	ViromeQC	2019	Sample-level contamination assessment	Quantifies nonviral contamination in VLP viromes	115	GitHub	https://github.com/SegataLab/viromeqc
**T26**	metaviralSPAdes	2020	Virus-specific SPAdes extension	Viral subgraph ID + completeness assessment; also used for contig verification	320	GitHub	https://github.com/ablab/spades
**T27**	VIRIDIC	2020	Nucleotide intergenomic similarity	ICTV-recommended algorithm for genus/species demarcation (also used in Taxonomy)	819	GitHub + Web	https://github.com/CristinaMoraru/VIRIDIC
**T28**	CheckV	2021	Genome quality assessment	5 quality tiers; 76 262 reference genomes; adopted by IMG/VR	1790	Bitbucket	https://bitbucket.org/berkeleylab/checkv
**T29**	Clinker	2021	Gene cluster comparison visualization	Automated comparison figures	1393	GitHub	https://github.com/gamcil/clinker
**T30**	pyGenomeViz	2022	Genome comparison visualization (Python)	Annotated comparative genomics figures	N/A	GitHub	https://github.com/moshi4/pyGenomeViz
**T31**	viralFlye	2022	Long-read viral assembly	Long-read metagenomics support	32	GitHub	https://github.com/Dmitry-Antipov/viralFlye
**T32**	vRhyme	2022	Viral contig binning	Coverage + nucleotide composition signals	113	GitHub	https://github.com/AnantharamanLab/vRhyme
**T33**	Phables	2023	Flow decomposition on assembly graphs	**49% more high-quality genomes than existing tools**	42	GitHub	https://github.com/Vini2/phables
**T34**	COBRA	2024	Paired-end extension of incomplete assemblies	Improves completeness and contiguity	46	GitHub	https://github.com/linxingchen/cobra
Gene annotation and functional prediction							
**No.**	**Tool**	**Year**	**Approach**	**Key metric/feature**	**Citations** **(Apr 2026)**	**Availability**	**URL**
**T35**	Prodigal	2010	Prokaryotic gene recognition	Fast; widely used general gene caller	13 001	GitHub	https://github.com/hyattpd/Prodigal
**T36**	pVOGs	2017	Prokaryotic virus orthologous groups	Viral marker gene detection; integrated into many pipelines	414	Web	https://ftp.ncbi.nlm.nih.gov/pub/kristensen/pVOGs/
**T37**	HHpred	2018	Profile-profile HMM comparison	Remote homology detection	2719	Web	https://toolkit.tuebingen.mpg.de/tools/hhpred
**T38**	PHANOTATE	2019	Dynamic programming for phage ORFs	Finds genes missed by Prodigal/GeneMarkS/Glimmer; handles overlapping frames	319	GitHub	https://github.com/deprekate/PHANOTATE
**T39**	DRAM-v	2020	Metabolic pathway annotation	Identifies auxiliary metabolic genes (AMGs)	1097	GitHub	https://github.com/WrightonLabCSU/DRAM
**T40**	PhANNs	2020	ANN structural protein classifier	F1 = 0.875 across 10 structural classes	113	GitHub + Web	https://github.com/Adrian-Cantu/PhANNs
**T41**	PHROGs	2021	HMM protein clustering (38,880 clusters)	50.6% functional annotation across 17 473 reference viruses	425	Web	https://phrogs.lmge.uca.fr/
**T42**	MultiPhATE2	2021	Parallel multi-gene-finder pipeline	Runs multiple gene finders simultaneously	24	GitHub	https://github.com/carolzhou/multiPhATE2
**T43**	Pharokka	2023	Integrated pipeline (PHANOTATE + PHROGs)	**Standard pipeline; <5 min for 50 kb genome**	519	GitHub	https://github.com/gbouras13/pharokka
**T44**	VPF-PLM	2024	Protein language model annotation	+29% annotated ocean virome protein families	95	GitHub	https://github.com/kellylab/viral-protein-function-plm
**T45**	ProstT5	2024	Protein sequence → 3Di structural alphabet	Bilingual sequence-structure translation	364	GitHub	https://github.com/mheinzinger/ProstT5
**T46**	Foldseek	2024	Ultrafast structural search	4–5 orders of magnitude faster than Dali/TM-align at comparable sensitivity	2365	GitHub + Web	https://github.com/steineggerlab/foldseek
**T47**	Empathi	2025	Hierarchical protein embeddings	2× on environment viromes (EnVhogDB); 3× on cultured phages	6	HuggingFace	https://huggingface.co/AlexandreBoulay/EmPATHi
**T48**	GOPhage	2025	Genomic context + transformer embeddings	+6.78% accuracy on divergent proteins	9	GitHub	https://github.com/jiaojiaoguan/GOPhage
**T49**	Phold	2026	Structure-informed (ProstT5 → Foldseek)	**>50% gene annotation versus ~35% homology-only**	30	GitHub	https://github.com/gbouras13/phold
Taxonomy and classification							
**No.**	**Tool**	**Year**	**Approach**	**Key metric/feature**	**Citations** **(Apr 2026)**	**Availability**	**URL**
**T50**	VICTOR	2017	Genome-BLAST Distance Phylogeny	Automated species/genus demarcation	699	Web	https://victor.dsmz.de/
**T51**	ViPTree	2017	Genome-wide tBLASTx proteomic trees	Viral proteomic tree server	1004	Web	https://www.genome.jp/viptree/
**T52**	vConTACT2	2019	Gene-sharing networks	96% genus-level ICTV agreement (pre-2022 framework)	990	Bitbucket	https://bitbucket.org/MAVERICLab/vcontact2
**T53**	PhaGCN	2021	Graph convolutional network + CNN	Semi-supervised classification	143	GitHub	https://github.com/KennthShang/PhaGCN
**T54**	PhaGCN2	2023	Extended PhaGCN (DNA + RNA viruses)	89.30% recall; 83.91% precision; applied to GPD and GOV2.0 datasets	112	GitHub + Web	https://github.com/KennthShang/PhaGCN2.0
**T55**	PhaBOX	2023	Integrated platform (PhaMer + PhaGCN + CHERRY + PhaTYP)	Unified ID + taxonomy from metagenomes (also used in host prediction)	117	GitHub + Web	https://github.com/KennthShang/PhaBOX
**T56**	GRAViTy-V2	2024	Composite generalized Jaccard distances	Genome relationship analysis	10	GitHub	https://github.com/Mayne941/gravity2
**T57**	taxMyPhage	2025	Automated genus/species classification	Aligned with current ICTV revisions; dsDNA phages	76	GitHub	https://github.com/amillard/tax_myPHAGE
**T58**	vConTACT3	2025	ML-based hierarchical classification	**>95% ICTV agreement (97.6% genus, 98.7% subfamily, 100% family/order)**	6	Bitbucket	https://bitbucket.org/MAVERICLab/vcontact3
**Lifestyle prediction**							
**No.**	**Tool**	**Year**	**Approach**	**Key metric/feature**	**Citations** **(Apr 2026)**	**Availability**	**URL**
**T59**	PHACTS	2012	Random Forest on protein similarity	99% precision (confident predictions); 88% overall sensitivity	305	GitHub	https://github.com/deprekate/PHACTS
**T60**	BACPHLIP	2021	HMM domains + Random Forest	98.3% accuracy on 423 independent test phages	294	GitHub	https://github.com/adamhockenberry/bacphlip
**T61**	DeePhage	2021	CNN on one-hot encoded DNA	89% accuracy; classifies contigs ≥100 bp	113	GitHub	https://github.com/shufangwu/DeePhage
**T62**	PhaTYP	2023	BERT pre-trained + fine-tuned	Outperforms prior methods on short contigs	206	GitHub + Web	https://github.com/KennthShang/PhaTYP
**T63**	DeepPL	2024	NLP on nucleotide sequences	94.65% accuracy	10	GitHub	https://github.com/Wu-Microbiology/DeepPL
**T64**	ProkBERT PhaStyle	2025	Genomic language models (21–26 M params)	**BA 0.88–0.93; MCC 0.75–0.86 on 500 bp fragments**	1	GitHub	https://github.com/nbrg-ppcu/PhaStyle
Anti-phage defence system detection							
**No.**	**Tool**	**Year**	**Approach**	**Key metric/feature**	**Citations (Apr 2026)**	**Availability**	**URL**
**T65**	CRISPRDetect	2016	Array detection + boundary refinement	CRISPR array identification	393	GitHub + Web	https://github.com/davidchyou/CRISPRDetect_2.4
**T66**	CRISPRCasFinder	2018	Integrated array + Cas protein ID	Combined array and Cas detection	1561	GitHub + Web	https://github.com/dcouvin/CRISPRCasFinder
**T67**	AcrFinder	2020	Homology + guilt-by-association + self-targeting spacers	Anti-CRISPR operon mining	80	GitHub + Web	https://github.com/HaidYi/acrfinder
**T68**	AcRanker	2020	XGBoost ranking	Identified AcrIIA20 and AcrIIA21	119	GitHub	https://github.com/amina01/AcRanker
**T69**	CRISPRCasTyper	2020	Automated subtype classification	98.6% accuracy	303	GitHub + Web	https://github.com/Russel88/CRISPRCasTyper
**T70**	PADLOC	2021/22	HMM + system completeness validation	Web server; customizable classifications	253	GitHub + Web	https://github.com/padlocbio/padloc
**T71**	DefenseFinder	2022	HMM profiles + MacSyFinder rule engine	60 families; 151 subtypes across 21 000 genomes	775	GitHub + Web	https://github.com/mdmparis/defense-finder
**T72**	AcrNET	2023	Deep learning anti-CRISPR prediction	Beyond homology-based methods	24	GitHub	https://github.com/banma12956/AcrNET
**T73**	AcaFinder	2023	Aca gene detection	Independent signal for novel anti-CRISPR loci	22	GitHub	https://github.com/boweny920/AcaFinder
**T74**	DefensePredictor	2025	Protein language model embeddings	42 novel defence systems validated across 69 *E. coli* strains	N/A	GitHub	https://github.com/Alextianyf/DefensePredictor
Host prediction							
**No.**	**Tool**	**Year**	**Approach**	**Key metric/feature**	**Citations (Apr 2026)**	**Availability**	**URL**
**T75**	HostPhinder	2016	k-mer similarity to reference DB	First dedicated host prediction tool	193	GitHub + Web	https://github.com/julvi/HostPhinder
**T76**	WIsH	2017	Markov models on host genomes	Up to 63% genus accuracy; 100× faster than alignment	317	GitHub	https://github.com/soedinglab/WIsH
**T77**	RaFAH	2021	Random Forest on 43 644 protein clusters	Consistent across RefSeq/SAG/metagenomic benchmarks	145	GitHub	https://github.com/felipehcoutinho/RaFAH
**T78**	HostG	2021	Graph convolutional network (semi-supervised)	GCN-based host prediction	59	GitHub	https://github.com/KennthShang/HostG
**T79**	SpacePHARER	2021	Protein-level CRISPR spacer matching	1.4–4× sensitivity over BLASTN at metagenomic scale	109	GitHub	https://github.com/soedinglab/spacepharer
**T80**	CHERRY	2022	Knowledge graph + graph convolutional encoder	Improved species-level accuracy	105	GitHub + Web	https://github.com/KennthShang/CHERRY
**T81**	PHIST	2022	k-mer-based alignment-free	+3–20 pp species accuracy; laptop-scale runtime	59	GitHub	https://github.com/refresh-bio/PHIST
**T82**	PHISDetector	2022	Unified CRISPR/prophage/similarity platform	Single platform for multiple interaction signals	52	GitHub + Web	https://github.com/HIT-ImmunologyLab/PHISDetector
**T83**	vHULK	2022	Neural network on viral protein family scores	Annotated genomic features input	36	GitHub	https://github.com/LaboratorioBioinformatica/vHULK
**T84**	iPHoP	2023	Integrated ensemble (homology + CRISPR + k-mer + ML)	**1.5–13× more predictions at equivalent FDR; >300 GB database**	413	Bitbucket	https://bitbucket.org/srouxjgi/iphop
**T85**	PhageHostLearn	2024	ESM-2 embeddings of RBP + receptor sequences	ROC AUC 81.8%; strain-level for *Klebsiella*	60	GitHub	https://github.com/dimiboeckaerts/PhageHostLearn
**T86**	PHPGAT	2025	Graph attention on heterogeneous knowledge graphs	Multimodal phage-host interaction prediction	14	GitHub	https://github.com/ZhaoZMer/PHPGAT
**T87**	PHIStruct	2025	SaProt protein structure embeddings	+7%–9% over sequence-only for divergent phages	20	GitHub	https://github.com/bioinfodlsu/PHIStruct
**T88**	MoEPH	2025	Gated Mixture-of-Experts (transformer + statistical)	Combines PLM embeddings with statistical descriptors	0	N/A	N/A
**T89**	RBPseg	2025	ESMFold + structural domain ID	First large-scale phage tail fibre structure atlas	3	GitHub	https://github.com/VKleinSousa/RBPseg
Integrated pipelines and structural resources							
**No.**	**Tool**	**Year**	**Description**	**Key feature**	**Citations (Apr 2026)**	**Availability**	**URL**
**T90**	BFVD	2025	Big Fantastic Virus Database	351 242 predicted viral protein structures; >62% novel	78	GitHub + Web	https://bfvd.foldseek.com/
**T91**	Sphae	2025	Snakemake workflow wrapping 12 tools	End-to-end processing in <10 min	14	GitHub	https://github.com/linsalrob/sphae

**Figure 2:**
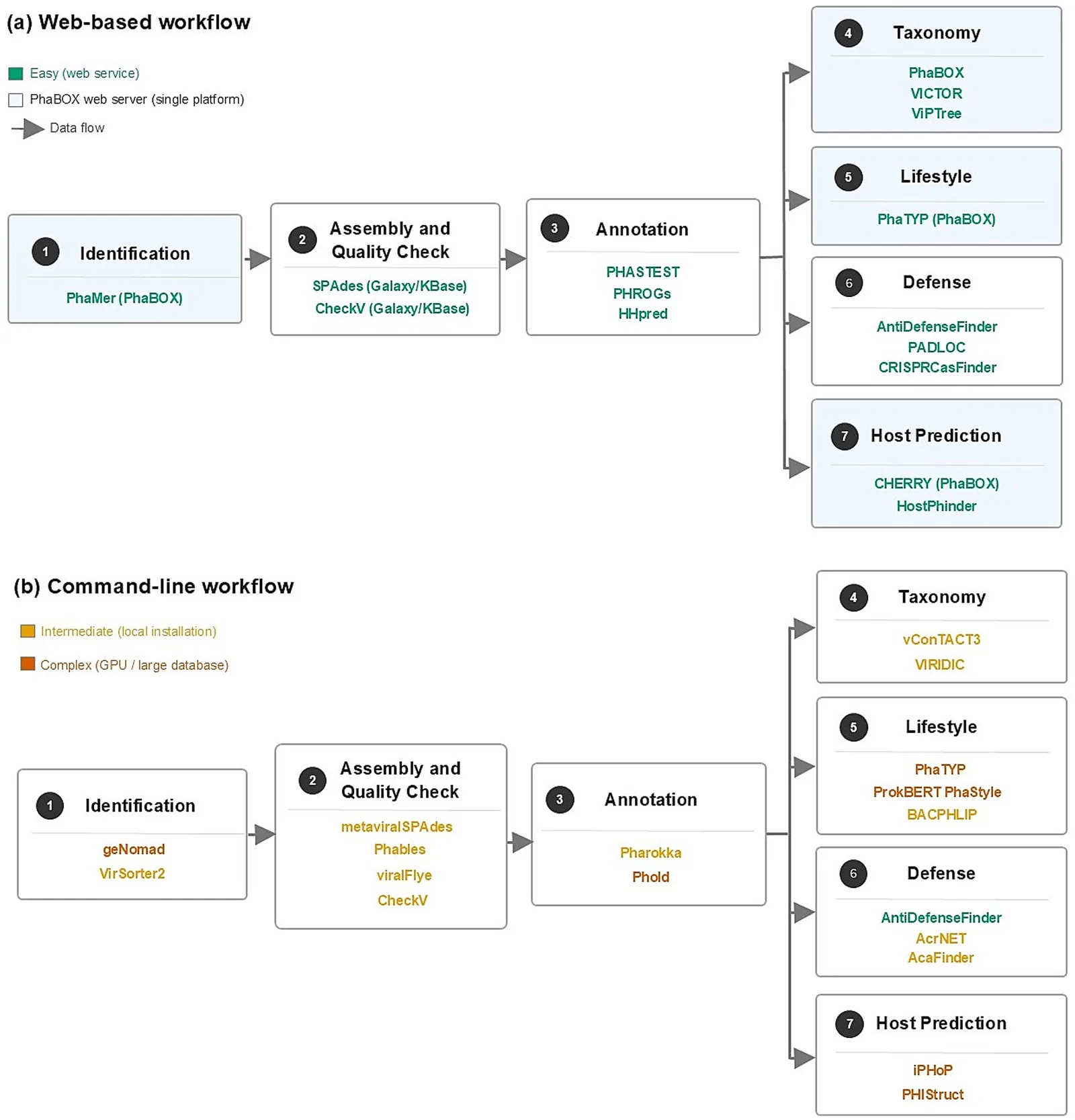
Proposed workflows for phage analysis of metagenomics data using (a) web-based or (b) command-line tools from identification through assembly/quality control and annotation to downstream analyses (taxonomy, lifestyle, defence systems, and host prediction), with data flow indicated between steps. Both workflows show analogous steps offering greater flexibility, scalability, and customization based on the resources available to run the tools.

Bold entries indicate the tool recommended as the primary choice for the corresponding workflow step in Fig. 2b and Table 3.

## Search strategy and scope

To ensure a comprehensive and reproducible evaluation of the computational landscape, literature and repository searches were conducted across PubMed, Google Scholar, bioRxiv, and GitHub up to March 2026, with specific computational tasks (e.g. identification, assembly). Inclusion criteria: dedicated phage bioinformatics tools (published from 2012 to 2026, including preprints) introducing novel algorithmic architectures or measurable performance advancements. Exclusion criteria: general-purpose computational utilities (e.g. BLAST, HMMER), which are discussed solely as infrastructural dependencies. Claude (Anthropic) was used to assist with data analysis during the preparation of this manuscript Performance metrics were extracted directly from original publications or independent benchmarking studies; no novel benchmarking was conducted for this review. However, because these metrics derive from different datasets, contig-length ranges, metric definitions, and evaluation protocols, they may not directly be comparable across tools.

## Phage sequence data and databases

Most computational phage tools depend on reference databases for training, validation, and comparison. The quality, completeness, and taxonomic framework of these databases can directly shape tool performance and set fundamental limits on what any algorithm can detect or classify. Five databases form the reference infrastructure for phage bioinformatics.

The NCBI Viral Genomes Resource curates complete viral genomes deposited in the International Nucleotide Sequence Database Collaboration (INSDC) databases and generates Reference Sequence (RefSeq) records for each recognized viral species [10]. Most phage identification and taxonomy tools use RefSeq entries as their training or reference database.

IMG/VR version 4 is the largest repository of uncultivated virus genomes, containing >15 million virus genomes and genome fragments derived from metagenomes, metatranscriptomes, and metaviromes a roughly six-fold increase over the previous version, driven by systematic application of geNomad for virus detection and CheckV for quality assessment [1]. IMG/VR applies current International Committee on Taxonomy of Viruses (ICTV) taxonomic standards and provides functional and ecological metadata within the Joint Genome Institute platform.

The Actinobacteriophage Database (PhagesDB) catalogues Actinobacteriophages discovered through the SEA-PHAGES programme and affiliated groups [11]. It has grown to >30 000 entries by 2026, with over 5000 fully sequenced genomes (accessed March 2026), the largest dedicated resource for Actinobacteriophage diversity.

INPHARED provides an automated pipeline for retrieving, curating, and analysing complete phage genomes from public repositories with structured metadata [2]. Beyond curation, INPHARED has also revealed systemic gaps: Cook *et al*. [2] identified severe sampling biases in the global collection, finding that 75% of all sequenced phages had been isolated on only 30 bacterial genera a bias that skews both database content and the training data available to computational tools. INPHARED is distributed through GitHub with automated updates.

The ICTV Virus Metadata Resource (VMR) provides the definitive list of exemplar viruses for every classified species, including accession numbers, genome composition, and host information [12]. Unlike the sequence-centric databases above, the VMR is a taxonomic reference used to align computational classification with official nomenclature. Following the 2022 ICTV reclassification, further discussed in the taxonomy and classification chapter, required databases and tools alike had to adopt the revised genomic nomenclature with INPHARED, IMG/VR, and vConTACT3 [13] now incorporating it natively.

Collectively, these resources are indispensable. They enable large-scale genomic and ecological landscape studies and serve as the foundation upon which downstream phage bioinformatics tools are developed. However, the pronounced sampling biases and taxonomic skew that currently characterize these databases, inevitably propagate into the training and evaluation of any tool built upon them. Expanding phage isolation and sequencing beyond the current 30 dominant host genera to uncover the true diversity is essential for improving tool generalizability.

## Phage identification and prophage detection

Phage identification within metagenomic datasets is the starting point for most downstream analyses. A typical environmental metagenome contains millions of contigs, mostly of cellular origin, with viral sequences as a minority fraction that must be computationally separated [14]. Many viral lineages share no detectable homology with characterized references given the diversity of phage genomes, so purely database-dependent approaches may miss them. Over the past decade, tools have progressed from reference-dependent homology searches through supervised machine learning to transformer-based foundation models (Fig. 1). Rather than relying on a single tool, analysts can combine multiple approaches tailored to their specific requirements to achieve better results (Fig. 2).

VirSorter was the first widely adopted homology era tool for mining viral signals from microbial genomic data, combining reference-based hallmark gene searches with enrichment metrics to achieve >95% recall on contigs of 10 kilobases or longer [3]. Its dependence on reference databases, however, meant sensitivity dropped on shorter contigs and novel phage lineages. Additional homology-era tools including MARVEL [15] extended detection using Random Forest classifiers trained on genomic features, resulting in better recall but remained constrained by database completeness.

**Figure 3:**
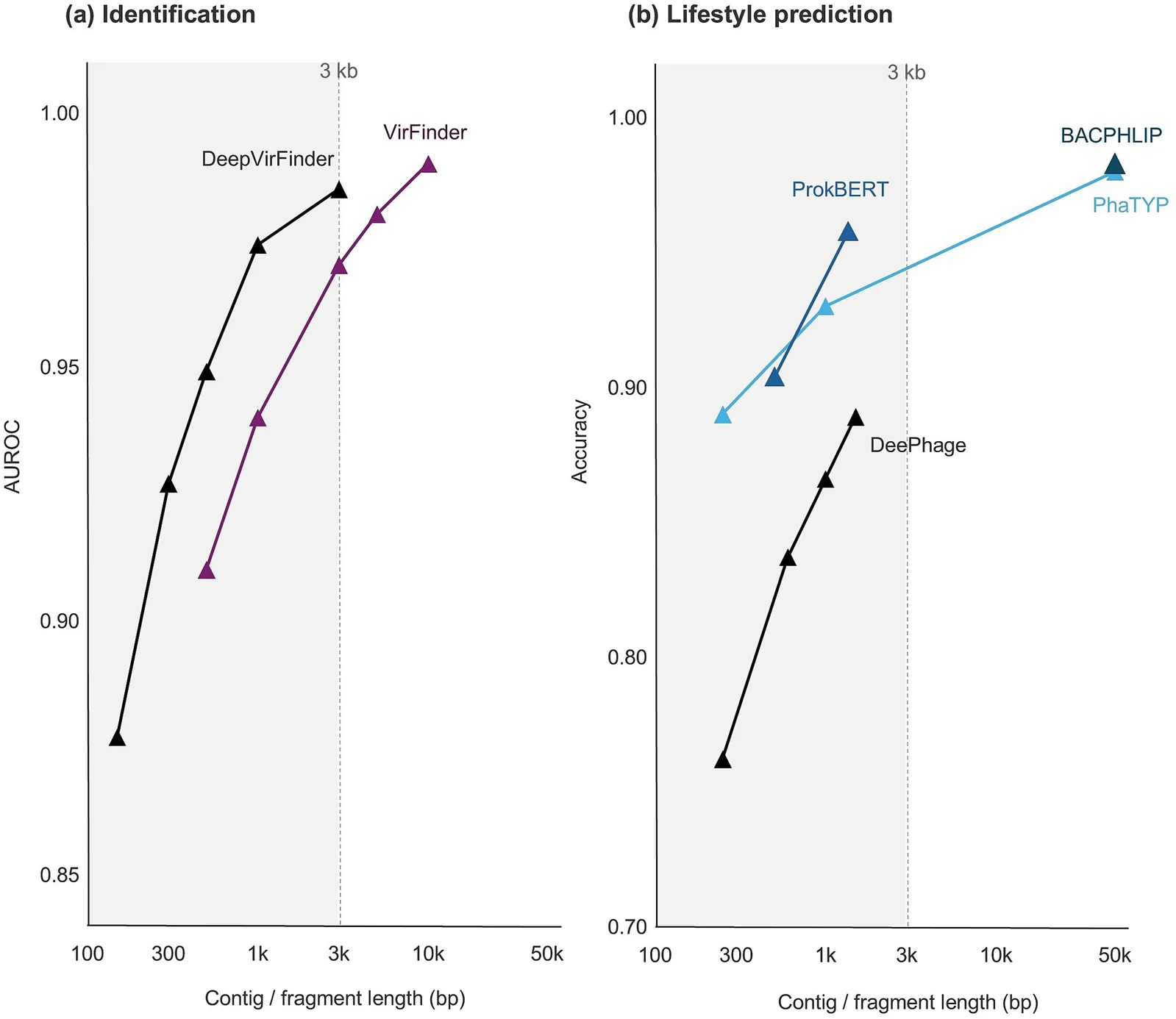
Performance of phage analysis models across contig lengths for (a) identification and (b) lifestyle prediction tasks. Area under the ROC curve (AUROC) for DeepVirFinder and VirFinder increases with contig/fragment length, with both models approaching high accuracy (>0.98) for sequences ≥3 kb. Classification accuracy for DeePhage, PhaTYP BACPHLIP, and ProkBERT improves with fragment length, with BACPHLIP and PhaTYP achieving the highest accuracies at longer contigs. Dashed vertical line indicates the 3 kb contig length threshold used for reliable prediction. All values are drawn from the primary publications cited in the main text.

VirFinder marked the shift from the homology era to the machine learning era, applying logistic regression to *k*-mer frequency signatures and achieving a 78-fold higher true-positive rate than VirSorter on 1 kilobase contigs at equivalent false-positive rates showing a considerable improvement on shorter contigs [5]. DeepVirFinder, its convolutional neural network extension, improved performance across all measured fragment lengths, reaching area under the receiver operating characteristic curve values of 0.93–0.98 for sequences of 300–3000 bp^16^ (Fig. 3a). Seeker adopted a Long Short-Term Memory architecture operating directly on raw DNA sequences without feature engineering, enabling alignment-free identification of phages with little similarity to known families [16]. Other notable tools in this era, PPR-Meta [17] and viralVerify [18] (see Genome  Assembly, Quality Assessment, and Comparative Genomics section), perform three-way classification of contigs as phage, plasmid, or chromosomal. VIBRANT [19] classifies contigs using HMM searches against viral protein families, and VirSorter2 [20] runs five random-forest classifiers in parallel, each specialized for a different viral group (dsDNA phages, nucleocytoplasmic large DNA viruses (NCLDVs), RNA viruses, ssDNA viruses, and virophages). Beyond these general-purpose identifiers, some tools target specific use cases: MetaPhinder [21] integrates BLAST hits across multiple reference phage genomes for sequence-level classification, while Kraken2, benchmarked by Ho *et al*. [14], prioritizes false-positive minimization (precision 0.96 in mock community evaluation), making it suited for studies where specificity matters more than sensitivity.

Transformer architectures entered phage identification in late 2022. PhaMer [22] and INHERIT [23] applied transformer and DNABERT-style [24] models, respectively, to phage contigs, with PhaMer achieving a 27% F1 score improvement over prior tools on mock metagenomic data. For this field of virus classifications, geNomad [7] shows the strongest reported overall performance, achieving a Matthews correlation coefficient (MCC) of 0.953 and an F1-score of 0.973, compared with an MCC of 0.813 for VirSorter2. geNomad uses a hybrid of alignment-free (IGLOO encoder) and >200 000 marker protein profiles to perform virus identification, plasmid detection, functional annotation, and taxonomic assignment within a single framework.

Prophage detection requires precise boundary detection within bacterial chromosomes rather than whole-contig classification. Classical homology-based tools include PhiSpy [25], which combines similarity and composition strategies to recover 94% of prophages (6% false-negative rate), and the widely used web server PHASTER [26] alongside its faster successor, PHASTEST [27]. For scalable analysis, DEPhT [28] offers multi-modal run settings (fast, normal, and sensitive) for targeted screening, extraction, and annotation. To circumvent the computational bottlenecks and database dependencies of these homology methods, tools like PhageBoost [29] provide a purely machine-learning-driven alternative. By evaluating viral genomic architecture, such as gene density, sequence composition, and operon direction, rather than sequence similarity, PhageBoost detects highly divergent prophages at execution speeds explicitly tailored for high-throughput bacterial whole-genome sequencing (WGS) pipelines.

Despite these reported accuracy gains, independent benchmarks [14, 30] reveal substantial tool-to-tool and dataset-to-dataset variability, with no single tool performing reliably across environments. Ho *et al*. found that 89.7% of true phage contigs were identified by more than half the tools tested, yet only 11.6% were detected by all 10 tools [14], indicating that individual tools capture distinct subsets of viral diversity and thorough detection requires combining multiple approaches. Performance of various tools is highly dataset-dependent: Wu *et al*. documented a highly variable true positive rates ranging from 0% to 97% across nine tools evaluated on eight datasets from three biomes [30], underscoring that no single tool performs reliably across all environments; the right tool needs to be judiciously selected for the right purpose and that combining multiple complementary approaches is essential for thorough phage detection.

## Genome assembly, quality assessment, and comparative genomics

Genome assembly is the checkpoint between identified viral contigs and biological interpretation. Incomplete or fragmented assemblies propagate errors into host prediction, lifestyle classification, and taxonomy [31]. Phage-specific obstacles such as terminal repeats, high mutation rates, uneven coverage, and mosaic genome architectures make phage assembly more challenging than bacterial assembly [18, 32], giving rise to a repertoire of quality check and assembly tools.

Tools from this category typically fall under the homology era starting with the generic assembler SPAdes [33] using multi-*k*-mer and a multiple de Bruijn graph framework for WGS. Its virus-specific extension metaviralSPAdes [18] is a homology-based tool that introduced modules for identifying viral subgraphs, distinguishing viral from bacterial contigs, and assessing genome completeness in metagenomic data. With the advent of long-read sequencing, new tools such as viralFlye [34] emerged for the assembly of long reads.

Post assembly refinement tools were also created to complement and boost genome assembly. COBRA [35] extends incomplete assemblies using paired-end information to generate longer and more complete assemblies. Phables [32] resolves tangled assembly graphs using flow decomposition to recover individual phage genomes through shared sequence regions. When assemblies remain fragmented, vRhyme [36] bins viral contigs into metagenome-assembled viral genomes (vMAGs) using coverage and nucleotide composition signals, providing the genome-level resolution required for most downstream analyses in viral metagenomics.

For viral genome quality assessment, CheckV [31] is the standard tool. Quality assessment is carried out through estimating completeness across five tiers (complete, high-quality, medium-quality, low-quality, and undetermined) and removing flanking host regions from provirus sequences against a database of 76 262 complete viral genomes. Its quality assessment has been adopted by IMG/VR. For sample-level quality control, ViromeQC [37] quantifies nonviral contamination in virus-like particle (VLP)-enriched viromes.

For comparative genomics and visualization, Clinker [38] and PyGenomeViz generate annotated genome comparison figures to visualize gene synteny analysis. Databases such as INPHARED [2] provide a continuously updated curated database supporting comparative analyses.

Early assembly workflows relied on general-purpose assemblers not optimized for viral genomes, often producing fragmented and incomplete contigs. The development of virus-specific modules within established frameworks (metaviralSPAdes), followed by phage-tailored approaches such as Phables, has progressively improved recovery rates. Quality assessment tools like CheckV now provide standardized completeness metrics that enable consistent evaluation across studies, though assembling complete genomes from short-read metagenomes remains difficult for phages with complex terminal structures.

Across the three-paradigm framework, assembly and quality assessment are the one domain where this progression has hardly occurred. The homology and classical-algorithm paradigm perform nearly all the work, spanning de Bruijn graph assembly (SPAdes, metaviralSPAdes, viralFlye), graph resolution and extension (Phables, COBRA), and reference-based completeness estimation (CheckV). Machine learning has contributed only at the margin of post-assembly binning, where vRhyme applies supervised neural-network and random-forest models to tetranucleotide and coverage signals to recover vMAGs. Foundation models remain absent because assembly is an exact combinatorial graph problem: protein and genomic language models excel at semantic pattern recognition but struggle with the Eulerian-path and de Bruijn graph resolution required to untangle fragmented assemblies caused by long terminal repeats and high mutation rates. The limitation may therefore be structural rather than merely unexplored, and whether newer architectures can recast genome recovery as a learnable problem remains untested.

## Gene annotation and functional prediction

When relying solely on sequence homology, up to 80% of genes in a typical phage genome cannot be assigned a functional role [39, 40], a phenomenon termed viral dark matter that reflects the deep evolutionary divergence of phage proteins from well-characterized cellular homologs. Annotation tools have progressed through the same three computational eras: homology-based database searches, machine learning classifiers, and foundation model approaches including structure-informed prediction.

Gene calling, the first step in any annotation workflow, remains rooted in classical algorithms. PHANOTATE [41] addresses the distinctive features of phage gene organization, overlapping reading frames, nonstandard start codons, and high gene density, using dynamic programming to evaluate every possible open reading frame. Across 2133 complete genomes, PHANOTATE consistently identified genes missed by Prodigal [42], GeneMarkS [43], and Glimmer [44], though those four callers agreed on 82% of predictions [41]. Prodigal remains widely used for its speed and larger predicted coding sequences and forms the basis of many downstream foundational tools (Table 2).

**Table 2:** Key dependencies of machine learning and deep learning tools.

**Category**	**Tool**	**Year**	**Architecture**	**Gene calling (Prodigal, pyrodigal, PHANOTATE, GeneMark, six-frame translation)**	**Homology search (BLAST, DIAMOND, HMMER, HMMscan, hmmsearch, MMseqs2)**	**Genome LM (DNABERT, DNABERT-2, ProkBERT, Nucleotide Transformer)**	**Protein LM (ESM-1b, ESM-2, ProtBERT, ProT5, ProtTrans, SaProt)**	**Structure tools (AlphaFold, ESMFold, Foldseek, ProstT5)**
**Identification**	**VirFinder**	2017	Logistic regression + *k*-mer	○	○	○	○	○
	**MARVEL**	2018	Random Forest	**●**	**●**	○	○	○
	**PPR-Meta**	2019	Bi-path CNN	○	○	○	○	○
	**DeepVirFinder**	2020	CNN on one-hot DNA	○	○	○	○	○
	**Seeker**	2020	LSTM on raw DNA	○	○	○	○	○
	**VIBRANT**	2020	NN + v-score metric	**●**	**●**	○	○	○
	**VirSorter2**	2021	Multi-classifier ML ensemble	**●**	**●**	○	○	○
	**PhaMer**	2022	Custom Transformer on protein tokens	**●**	**●**	○	○	○
	**INHERIT**	2022	DNABERT	○	○	**●**	○	○
	**geNomad**	2023	IGLOO encoder + CRF	**●**	**●**	○	○	○
**Annotation**	**PhANNs**	2020	ANN on amino-acid features	○	○	○	○	○
	**VPF-PLM**	2024	ESM-2 classifier	**●**	**●**	○	**●**	○
	**Empathi**	2025	Hierarchical PLM embeddings	**●**	**●**	○	**●**	○
	**GOPhage**	2025	ESM-2 + genomic-context Transformer	○	○	○	**●**	○
	**Phold**	2026	ProstT5 - > Foldseek structure-informed	**●**	**●**	○	○	**●**
**Taxonomy**	**vConTACT2**	2019	Gene-sharing network	**●**	**●**	○	○	○
	**PhaGCN**	2021	GCN + CNN	○	**●**	○	○	○
	**PhaGCN2**	2023	Extended GCN (DNA + RNA)	**●**	**●**	○	○	○
	**PhaBOX**	2023	Integrated DL platform	**●**	**●**	○	○	○
	**vConTACT3**	2025	Hierarchical ML classifier	**●**	**●**	○	○	○
**Lifestyle**	**PHACTS**	2012	Random Forest + FASTA similarity	○	**●**	○	○	○
	**BACPHLIP**	2021	RF on HMMER3 protein domains	○	**●**	○	○	○
	**DeePhage**	2021	CNN on one-hot DNA	○	○	○	○	○
	**PhaTYP**	2023	Custom BERT on protein tokens	**●**	**●**	○	○	○
	**DeepPL**	2024	DNABERT fine-tune	○	○	**●**	○	○
	**ProkBERT PhaStyle**	2025	Genomic language model	○	○	**●**	○	○
**Defense**	**AcRanker**	2020	XGBoost ranking	○	○	○	○	○
	**AcrNET**	2023	Deep NN + ESM-1b	○	○	○	**●**	○
	**DefensePredictor**	2025	PLM embeddings	○	**●**	○	**●**	○
**Host prediction**	**HostG**	2021	GCN	**●**	**●**	○	○	○
	**RaFAH**	2021	RF on 43 644 protein clusters	**●**	**●**	○	○	○
	**CHERRY**	2022	Graph encoder–decoder	**●**	**●**	○	○	○
	**vHULK**	2022	NN on viral protein families	○	**●**	○	○	○
	**iPHoP**	2023	Ensemble ML (5 methods)	**●**	**●**	○	○	○
	**PhageHostLearn**	2024	ESM-2 + XGBoost	**●**	**●**	○	**●**	○
	**MoEPH**	2025	Gated MoE of ProtBERT + ProT5	○	○	○	**●**	○
	**PHIStruct**	2025	SaProt (structure-aware PLM)	○	○	○	**●**	○
	**PHPGAT**	2025	Graph attention on multimodal KG	**●**	**●**	○	○	○
	**RBPseg**	2025	ESMFold + structural domain ID	○	○	○	○	**●**

In the homology era, functional annotation relied on searching predicted genes against curated reference databases. PHROG [45] provides 38 880 protein clusters from 868 340 proteins across 17 473 reference prokaryotic viruses, with 5108 functionally annotated clusters spanning nine categories via HMM profile-profile comparisons. These clusters serve as the primary annotation reference that tools such as Pharokka [46] query when assigning gene functions. Pharokka [46] has become the standard annotation pipeline, integrating gene calling (PHANOTATE or Prodigal), PHROGs assignment, and full functional annotation into a single tool that processes a 50 kb phage genome in under 5 min. Beyond Pharokka, alternative annotation pipelines include MultiPhATE2 [47], with the ability to run multiple gene finders in parallel for more comprehensive gene calling, and DRAM-v [48], which identifies auxiliary metabolic genes, phage-encoded genes that function within host metabolic pathways, during infection. Tools like HHpred [49] also extend homology-based annotation through profile–profile HMM comparisons, enabling remote homology detection for highly diverged proteins. Additionally, the availability of the pVOGs database [50] offers a complementary orthologous groups framework, widely used for viral marker gene detection and integrated into numerous downstream annotation and identification pipelines.

Machine learning classifiers brought supervised approaches to specific annotation tasks. PhANNs [51] classifies phage proteins into 10 structural classes such as major capsid, tail fibre, and baseplate using an ensemble of artificial neural networks (weighted F1 = 0.875), enabling structural annotation of proteins that lack detectable homologs. Other tools such as PhageScanner [52] adopt similar machine learning strategies for phage protein classification.

Protein language models marked the shift to the foundation model era for annotation. VPF-PLM [53] uses mean-pooled embeddings from a pretrained protein language model to classify viral proteins into nine PHROGs functional categories via a feedforward neural network, enabling alignment-free annotation of proteins beyond the reach of profile HMMs. Applied to the EFAM ocean virome database, VPF-PLM annotated 26 770 previously unannotated protein families, a 29.4% increase over existing annotations. Two 2025 tools extend this approach: Empathi [54] reorganizes PHROGs into 44 hierarchical functional categories and trains binary classifiers on protein embeddings to double annotation rates relative to homology-only methods from 16% to 33%, and GOPhage [55] adds genomic context by using a Transformer to capture protein order along phage contigs, improving annotation of proteins lacking database homology.

A notable recent addition to the field, Phold [8], translates amino acid sequences into the 3Di structural alphabet via ProstT5 [56] and subsequently searches these representations against over 1.36 million predicted phage protein structures using Foldseek [57]. This pipeline annotates >50% of genes on a typical phage genome, compared with ~35% for homology-only methods such as Pharokka (Table 1). Despite these improvements, structure-informed annotation leaves roughly half of viral genes uncharacterized, a ceiling dictated by biophysics rather than computational limits. Many phage proteins feature intrinsically disordered regions (IDRs) [58] or require physical interaction with host complexes to adopt functional folds. While monomeric prediction algorithms remain blind to host-dependent conformational dynamics, generalized models like AlphaFold 3 [59] shift this paradigm. By accurately modelling multimolecular complexes, they provide a theoretical path to resolve viral folds dependent on host interactions. The primary bottleneck consequently shifts from algorithmic limitation to computational scalability, requiring the field to adapt these resource-intensive models for high-throughput viromics.

## Taxonomy and classification

After gene annotation, the next step is placing phages within a coherent taxonomic framework. The 2022 ICTV reclassification abolished the morphology-based families *Myoviridae*, *Siphoviridae,* and *Podoviridae*, replacing them with genomically defined taxa under the class *Caudoviricetes* [60]. Morphological classification could not scale to metagenomic phages lacking electron micrographs, and genomic analyses had revealed all three families were polyphyletic. New computational tools were needed to assign phages within this revised framework (Table 1). These tools have progressed from sequence-based distance metrics through gene-sharing networks to deep learning classifiers capable of hierarchical classification across the full ICTV hierarchy.

Early approaches in this space relied on sequence-based distance metrics, which remain valuable for targeted analyses but face scalability limits. VICTOR [61] applies Genome-BLAST Distance Phylogeny to produce phylogenomic trees with automated species and genus demarcation. ViPTree [62] generates viral proteomic trees from genome-wide tBLASTx similarities. VIRIDIC [63] implements the ICTV-recommended algorithm for nucleotide-based intergenomic similarities used in formal taxonomic proposals. These alignment-based tools remain essential for targeted phylogenetic analyses but scale poorly to tens of thousands of genomes.

Several additional homology-based tools address specific taxonomic needs: taxMyPhage provides automated genus and species-level classification of dsDNA phages aligned with current ICTV revisions [64], while GRAViTy-V2 applies composite generalized Jaccard distances built from generalized Jaccard similarity indices for genome relationship analysis [65]. vConTACT2 addressed scalability by pioneering the gene-sharing network paradigm, connecting phage genomes by shared protein clusters, and replicating 96% of existing genus-level ICTV assignments [4]. vConTACT2 was the methodological ancestor of the machine learning (ML)-era taxonomy tools, but the tool itself runs on classical homology and graph algorithms, classified only at the genus level, and left highly divergent phages in unresolved outlier clusters.

Deep learning integrated with network representations enabled classification under the new ICTV hierarchy. PhaGCN [66] combined convolutional neural network (CNN)-learned DNA features with protein similarity in a semi-supervised graph convolutional network. PhaGCN2 extended this to DNA and RNA viruses, achieving 89.30% recall and 83.91% precision on newly added ICTV2021 reference genomes (with the model trained on ICTV2020), and was subsequently applied at scale to classify the Gut Phage Database (142 809 sequences) and GOV2.0 datasets. PhaBOX [67] consolidates PhaGCN with phage identification (PhaMer), lifestyle prediction (PhaTYP), and host prediction (CHERRY) into a single web server, delivering family-level taxonomic classification alongside the other analyses through one interface. An updated PhaBOX2 release has since been formally published [68] as a major upgrade that broadens the platform’s identification, taxonomic, and host-prediction scope beyond bacteriophages to archaeal and eukaryotic viruses under the ICTV 2024 taxonomy. Its redesigned end-to-end workflow adds rigorous quality control, automatic removal of host contamination, clustering of sequences into viral operational taxonomic units (vOTUs), and marker-gene phylogenetic analysis. Departing from the ‘black-box’ character of purely deep-learning classifiers, PhaBOX2 adopts a ‘glass-box’ design that couples alignment-based strategies with machine-learning models to expose interpretable intermediate evidence alongside its predictions, and, running on dedicated high-performance infrastructure, delivers a fully automated pipeline.

Among currently available tools, vConTACT3 provides the broadest hierarchical taxonomy coverage, employing machine-learning-based hierarchical classification from genus to order across four out of six officially recognized viral realms [13]. Evaluated on prokaryotic viruses across four realms, vConTACT3 achieved >95% agreement with official ICTV taxonomy: 97.6% at genus, 98.7% at subfamily, and 100% at both family and order levels.

Together, these tools span the range from single-genome phylogenetics to large-scale automated classification, though all face the fundamental challenge of assigning consistent taxonomy to mosaic genomes where different genomic regions yield conflicting signals. Pervasive genome mosaicism remains a fundamental challenge. Smug *et al*. found extensive domain-level mosaicism across 133 574 representative phage proteins, with homologous domains shared across distinct functional classes in 45 of 101 categories [69]. Conflicting taxonomic signals across mosaic genomes expose a fundamental algorithmic flaw: enforcing strict hierarchical taxonomies onto reticulate, nonvertical evolutionary histories. Hierarchical models are mathematically misaligned with the pervasive horizontal gene transfer defining phage evolution. Future taxonomic algorithms might therefore shift from forced hierarchical clustering towards directed acyclic graphs or network-based models that natively resolve reticulate events.

## Lifestyle prediction

Phages follow several replication strategies, though two dominate current classification: the lytic cycle, in which the phage replicates and lyses the host cell, and the lysogenic cycle, in which it integrates as a prophage. Other strategies exist: chronic infection, pseudolysogeny, and carrier states [70], but computational tools have focused mainly on the lytic–lysogenic distinction. Distinguishing lytic from temperate phages is particularly important for clinical applications. Temperate phages carry inherent therapeutic risks, mediating horizontal gene transfer, mobilizing toxin genes, and conferring antibiotic resistance, so phage therapy candidates should be confirmed as obligately lytic before clinical use. Lifestyle prediction tools have progressed through two computational eras: supervised machine learning on engineered features, followed by genomic language models fine-tuned from foundation model pretraining.

Early supervised learning approaches to lifestyle prediction were constrained by small training sets and complete-genome requirements. Phage Classification Tool Set (PHACTS) [71], the first dedicated tool, combined protein similarity with a Random Forest classifier using a leave-one-out approach across 227 labelled phages, achieving 99% precision on 199 confident predictions from complete genomes (overall sensitivity 88%) with accuracy declining on partial proteomes. BACPHLIP [70] improved on this by feeding HMMER3-detected lysogeny-associated domain profiles, dominated by integrases and recombinases, to a Random Forest, reaching 98.3% accuracy on 423 independent test phages versus 79% for PHACTS; however, it operates only on complete genomes and cannot evaluate fragmented contigs (Fig. 3b). DeePhage [72] adopted a CNN architecture with one-hot encoded DNA, achieving accuracies from 76.2% on 100–400 bp fragments to 88.9% on 1200–1800 bp fragments, the first tool to classify contigs only a few hundred base pairslong.

Foundation models shifted the field to pretrained genomic language models fine-tuned for lifestyle classification. PhaTYP [73] adapts BERT through masked language model pretraining on RefSeq phage proteins before fine-tuning on the 1867-phage DeePhage benchmark (1290 virulent, 577 temperate), maintaining 0.89 accuracy on 100–400 bp fragments where DeePhage scored 0.76 and BACPHLIP could not run (Fig. 3b) DeepPL [74] instead fine-tunes DNABERT on 6-mer tokenised nucleotide sequences, achieving 94.65% accuracy.

ProkBERT PhaStyle [75] benchmarked three genomic language models: ProkBERT, DNABERT-2 [76], and Nucleotide Transformer on fragmented phage sequences; ProkBERT-mini (~20 million parameters) achieved balanced accuracy of 0.88–0.93 and MCC of 0.75–0.86 on 500 bp fragments, outperforming the substantially larger DNABERT-2 (117 M parameters) and Nucleotide Transformer (50–500 M), indicating that domain-specific pretraining matters more than model scale for this task. Figure 3b captures this generational shift: BACPHLIP’s 98.3% complete-genome accuracy cannot extend to short contigs, DeePhage climbs gradually from 0.76 to 0.89 with increasing length, while PhaTYP and ProkBERT PhaStyle maintain 0.88–0.94 accuracy across the full-length range, a move from tools requiring complete genomes to foundation models built for metagenomic fragments.

Training data remain scarce: ~1867 labelled examples constitute the primary benchmark dataset [73], and expansion requires labour-intensive experimental confirmation. The virulent-temperate binary inherently oversimplifies phage biology. To accurately model ecologically significant strategies such as pseudolysogeny, chronic infection, or carrier states, future foundation models must evolve from outputting discrete binary labels to generating continuous probability distributions. This ‘soft classification’ approach would mathematically capture the dynamic spectrum of phage lifecycles.

## Defence and counter-defence system prediction

Prokaryotic genomes encode at least 60 families of antiviral defence systems encompassing 151 subtypes identified by 2022 [77], well beyond the restriction–modification and CRISPR-Cas pathways identified through early molecular work. The current DefenseFinder catalogue spans these families, including abortive infection systems, retrons, cyclic oligonucleotide signalling pathways, and dozens of mechanistically uncharacterised systems [77]. These defence genes cluster within genomic defence islands, hotspots where multiple systems co-localize, often flanked by mobile genetic elements enabling horizontal transfer.

Defence-system detection relies on HMM profiles combined with architectural rules. CRISPR-specific tools established the earliest computational infrastructure: CRISPRDetect [78] for array detection and boundary refinement, CRISPRCasFinder for integrated array and Cas protein identification [79], and CRISPRCasTyper [80] complements this by assigning subtypes directly from repeat sequences using a machine-learning classifier, useful for orphan arrays lacking adjacent Cas genes. DefenseFinder identifies antiviral systems using curated HMM profiles combined with MacSyFinder, a rule engine enforcing synteny and gene co-occurrence constraints [77]. Applied to 21 000 prokaryotic genomes, it provided the first systematic view of the antiviral arsenal, initially detecting 60 families with more than 150 subtypes, with regular updates expanding coverage. PADLOC takes a complementary approach, employing HMM-based searches followed by system completeness validation through gene presence–absence and synteny criteria [81]. Its web server allows customizable classifications incorporating newly described systems [82]. Running both tools on the same dataset provides broader coverage than either alone. Millman *et al*. [83] combined computational prioritization with experimental phage-challenge plaque assays, confirming 21 new defence systems from 45 tested candidates and illustrating the pace at which the repertoire expands.

Counter-defence prediction has progressed through the three eras. Homology-based tools target anti-CRISPR (Acr) proteins through combined signal integration. AcrFinder [84] combines homology search, guilt-by-association via Aca-Aca operons, and self-targeting spacer detection, while AcaFinder [85] targets conserved Aca genes via HMM profiles or guilt-by-association with Acr homologs.

The machine-learning era brought AcRanker [86], which uses XGBoost on amino acid *k*-mer composition to rank anti-CRISPR candidates, successfully identifying AcrIIA20 and AcrIIA21 as inhibitors of *Streptococcus iniae* Cas9 and SpyCas9. Foundation-model approaches now extend beyond homology using protein language model embeddings. AcrNET [87] feeds ESM-1b embeddings alongside RaptorX structural and POSSUM evolutionary features into a CNN-based classifier to predict anti-CRISPR proteins. DefensePredictor [88] builds a gradient-boosting classifier on ESM2 embeddings to flag candidate defensive proteins including those with no detectable homology to known immune genes. It was applied across 69 *Escherichia coli* strains and 42 previously unknown systems experimentally validated through phage-challenge plaque assays.

Across the three paradigms, the two halves of this area sit at different stages of development. Defence-system detection remains firmly in the homology paradigm, built on curated HMM profiles enforced by syntenic rule engines (DefenseFinder with MacSyFinder, PADLOC), with machine learning entering only narrowly through CRISPRCasTyper’s repeat-based subtype classifier. This design imposes a hard ceiling because profile and rule-based methods recover only systems already represented in their catalogues. Counter-defence prediction, in contrast, spans all three eras, progressing from homology and guilt-by-association (AcrFinder, AcaFinder) through machine learning (AcRanker) to protein-language-model embeddings (AcrNET). Foundation models are now beginning to lift the detection ceiling as well: DefensePredictor couples ESM2 embeddings with a gradient-boosting classifier to surface candidate immune systems that share no detectable homology with known defences, extending discovery beyond CRISPR and curated profiles. The limitation common to both halves is experimental rather than algorithmic with candidates from either approach still require phage-challenge validation, the global defence landscape remains substantially under-sampled, and structure-informed anti-defence discovery through Foldseek is a plausible next frontier.

## Host prediction and phage–bacteria interaction

After identification, assembly, annotation, and taxonomic placement, the next question is which bacterial hosts a given phage can infect; this determination that is critical for phage ecology, therapeutic candidate selection, and interpretation of the millions of uncultured phage sequences catalogued in environmental and clinical metagenomes.

Host prediction draws on four signals from phage-host co-evolution: sequence homology, CRISPR spacer matching, codon usage bias, and metagenomic co-occurrence. Tools have progressed from single-signal methods through multi-feature machine learning to protein language model approaches.

In the homology era, early tools each targeted a single signal. HostPhinder [89] used *k*-mer similarity against a reference database of 2196 phages with known hosts, while WIsH [90] trained Markov models on host genomes, operating hundreds of times faster than alignment-based methods. SpacePHARER [91] enables protein-level CRISPR spacer matching with 1.4–4× greater sensitivity than BLASTN at metagenomic scale. These single-signal tools set accuracy ceilings that multi-feature integration subsequently raised.

The machine learning era enabled integration of multiple genomic features into unified prediction frameworks. RaFAH [92] applies a Random Forest trained on scores from 43 644 protein clusters, performing consistently across RefSeq, single-amplified genome, and metagenomic benchmarks. CHERRY [93] formulated host prediction as link prediction in a knowledge graph, using a graph convolutional encoder over protein and DNA features to substantially improve species-level accuracy. Several complementary tools extended coverage through alternative architectures: HostG [94] introduced graph convolutional networks for semi-supervised prediction; vHULK [95] feeds alignment scores against curated viral protein families into a neural network; and PHIST [96] improved species-level accuracy by 3% over alignment-based tools while running two orders of magnitude faster, suitable for routine use on a standard laptop. PHISDetector [97] unified detection of CRISPR spacers, prophage regions, and sequence similarity into a single ML-scored prediction pipeline.

iPHoP [98] addressed the fragmentation of these approaches by integrating BLAST homology, CRISPR spacer matching, three *k*-mer/composition tools (WIsH, VirHostMatcher, PHP), and RaFAH phage-protein content through a random-forest ensemble, providing 1.5–13× more host predictions. This gain confirms that combining orthogonal signal types yields greater coverage than refining any single approach. iPHoP’s full reference database, however, requires >300 GB of storage, contrasting sharply with PHIST’s laptop-scale runtime, a practical divide between thorough-but-heavy and fast-but-narrow tools that recurs across phage bioinformatics.

Foundation model approaches now extend this frontier by replacing or supplementing engineered features with protein language model embeddings. PhaBOX [67] consolidates CHERRY, PhaTYP, PhaMer, and PhaGCN into a unified web server-based platform; PHPGAT [99] applies GATv2 graph attention networks over a multimodal heterogeneous phage-host knowledge graph. PHIStruct [100] applies SaProt protein structure embeddings (650M parameters) to achieve F1 gain over sequence-only machine learning methods, and 5%–6% over BLASTp, specifically on phages where the training–test sequence similarity falls below 40%; and MoEPH [101] combines ProtBERT and ProT5 embeddings with statistical sequence descriptors through a gated Mixture-of-Experts fusion mechanism.

Genome-level prediction identifies likely host taxa but not the molecular determinants of specificity: the receptor-binding proteins (RBPs) on phage tail fibres. RBPseg [102] combines monomeric ESMFold predictions with structural domain identification and AF2M refinement to produce the first large-scale phage tail fibre structure atlas of 67 fibres grouped into 16 structural classes, validated by cryo-EM of five fibres from three phages.

PhageHostLearn [103] pushes towards strain-level resolution by predicting phage–host interactions directly from ESM-2 protein language model embeddings of RBP and bacterial receptor sequences, achieving a cross-validated receiver operating characteristic area under curve (ROC AUC) of 81.8% *in silico* (79.3% in laboratory validation). Applied to Klebsiella phage–host specificity, it shows strain-level prediction is tractable, though extension across genera requires further work. For host prediction, the practical question is what taxonomic resolution tools can reach. Most whole-genome predictors resolve the host only to genus or species, which is sufficient for ecological surveys and host-range screening. The strain and receptor-level resolution needed to select a therapeutic phage is so far achieved only by emerging receptor-binding-protein and receptor-based models, and only for individual pathogens such as Klebsiella (Supplementary Table 2).

Most tools still operate at the genus or species level. Strain-level prediction remains limited to single bacterial genera, all supervised methods depend on reference database completeness, and ensemble approaches carry computational costs that limit adoption in resource-constrained settings. While protein language models demonstrate sequence-level tractability for predicting interactions between receptor-binding proteins and host receptors, they abstract away the biophysics of viral infection. Strain-level specificity is a 3D thermodynamic process governed by dynamic binding affinities, not just sequence matching. To achieve clinical utility, next-generation predictors must integrate language-model embeddings with physics-based molecular docking and dynamics (MD) simulations to resolve precise receptor–ligand kinetics, as well as multi-omic models that compute the host’s intracellular defence landscape.

## Proposed workflows for phage characterization

The preceding sections reviewed >80 tools spanning eight categories. This breadth creates a practical selection problem: which tools should a researcher run, in what order, and with what alternatives? We propose two reference workflows calibrated to computational expertise using either web-based interfaces or command line interfaces (Fig. 2). Additional tools are also included to augment and increase the sensitivity and output. Table 3 distils these choices into a step-by-step decision guide, pairing each workflow stage with its recommended web-based and command-line tools, expected input and output, the best-suited use scenario indicating when to choose each option, and the principal caveat with its indicative computational tier. Supplementary Table 3 complements this with the computational resources, interface, compute tier, runtime, memory, database size, and graphics processing unit (GPU) needs that are required by every tool in both workflows.

**Table 3:** Decision guide to the proposed phage characterization workflows (Fig. 2).

**Workflow step (** Fig. 2 **)**	**Input**	**Web tool (** Fig. 2a **)**	**Command-line tool (** Fig. 2b **)**	**Main output**	**Best-suited scenario/when to choose**	**Key caveat and computational tier**
**1. Identification**	Assembled contigs or raw metagenome	PhaMer (PhaBOX)	geNomad (primary); VirSorter2 or VIBRANT	Putative viral contigs with confidence scores	First-pass viral discovery on any contigs. Run two or more tools when recall matters (novel or low-abundance phages); a single web pass (PhaMer/PhaBOX) suffices for a quick look.	**Browser/laptop–server.** Low inter-tool concordance; geNomad’s mixed output formats must be parsed before downstream steps.
**2. Assembly and quality check**	Quality-filtered reads (short or long)	SPAdes + CheckV (Galaxy, KBase)	metaviralSPAdes or viralFlye; Phables; CheckV	Assembled phage genomes with completeness and contamination estimate	Read type decides the assembler: Illumina short reads → metaviralSPAdes; ONT/PacBio long reads → viralFlye. Use Phables to bridge fragmented metagenome assemblies.	**Laptop–server (memory scales with data).** Complete genomes remain hard to recover from short-read metagenomes with complex terminal repeats.
**3. Annotation**	Phage genome/predicted ORFs	PHROGs + HHpred; PHASTEST (prophage)	Pharokka, then Phold; DRAM-v (AMGs)	Functional gene annotations	Fast homology pass on a laptop → Pharokka. When many genes stay hypothetical and a GPU is available → add Phold (structure-informed). Auxiliary-metabolic-gene survey → DRAM-v.	**Pharokka laptop; Phold GPU.** About half of genes stay hypothetical even with structure; Phold’s ProstT5 step needs a GPU (~66 min for 1419 genomes (<1 min per genome) on an NVIDIA A100).
**4. Taxonomy**	Phage genome(s)	PhaGCN (PhaBOX); VICTOR; ViPTree	vConTACT3; VIRIDIC	ICTV taxonomic ranks/genome clusters	Large sets → vConTACT3 gene-sharing clusters. A few genomes or formal ICTV proposals → VIRIDIC intergenomic identity and VICTOR/ViPTree trees.	**Laptop–server.** Anchored to ICTV-labelled references (>95% genus agreement on characterized genomes); highly divergent phages fall into unassigned clusters.
**5. Lifestyle**	Phage contig or genome	PhaTYP (PhaBOX)	PhaTYP or ProkBERT PhaStyle; BACPHLIP	Virulent versus temperate call	Contig length decides: short fragments or partial genomes → PhaTYP or ProkBERT PhaStyle; complete genomes → BACPHLIP.	**Laptop.** Output is virulent/temperate only; genome-trained tools lose accuracy on very short or partial inputs.
**6. Defence**	Phage or bacterial genome	DefenseFinder; PADLOC; CRISPRCasFinder	AntiDefenseFinder; PADLOC; CRISPRCasTyper; AcrNET/AcaFinder	Defence/anti-defence systems; CRISPR arrays and subtypes	Many genomes → DefenseFinder (laptop-scale; also gives anti-defence via AntiDefenseFinder). Few genomes, or CRISPR arrays and ncRNAs wanted in one run → PADLOC. Different databases, never benchmarked head-to-head → run both when completeness matters. CRISPR subtypes → CRISPRCasTyper; anti-CRISPR → AcrNET/AcaFinder.	**Laptop.** HMM- and rule-based, so only catalogued systems are detected; novel systems are missed.
**7. Host prediction**	Phage genome or contig	CHERRY (PhaBOX); HostPhinder; PHISDetector	iPHoP; PHIStruct	Predicted host taxonomy (usually genus)	Research goal decides resolution. Ecological/host-range surveys (genus) → iPHoP; low sequence similarity → PHIStruct (structure-aware, still genus). Strain-level therapeutic matching needs pathogen-specific receptor models (a current gap; see Supplementary Table 2).	**Browser; iPHoP HPC.** Reliable mainly to genus, not strain; iPHoP’s reference database exceeds 300 GB (~12 min per 5 genomes).

Bold type marks the workflow step label in the first column and, in the final column, the minimum computational tier required to run the recommended tools for that step.

Browser-accessible platforms enable end-to-end phage analysis without local installation, lowering barriers for noncomputational users (Fig. 2a). Although no standalone phage-specific web assembler exists, general-purpose platforms such as Galaxy (https://usegalaxy.org) and KBase (https://kbase.us) provide integrated environments for assembly and quality check using SPAdes [33] and CheckV [31]. For downstream analysis, PhaBOX [67] acts as the central hub (shaded boxes in Fig. 2a), integrating phage identification (PhaMer), taxonomy (PhaGCN), lifestyle prediction (PhaTYP), and host prediction (CHERRY) within a unified web interface. Specialized tools on other web browsers complement this workflow by providing additional analysis that may be required for more specific use cases. Prophage regions can be identified using PHASTEST [27] (phastest.ca). Functional annotation is supported by the PHROGs [45] database (using HMM-based clustering) with HHpred [49] for remote homology. For taxonomy analysis beyond PhaBOX, VICTOR [61], and ViPTree [62] provide phylogenomic and proteomic tree reconstruction via web servers. Defence system detection is supported by DefenseFinder [77] and PADLOC [81, 82], which provide complementary HMM-based platforms for the detection of anti-defence systems, while CRISPRCasFinder [79] and CRISPRCasTyper [80] enable CRISPR array detection and subtype classification. Host prediction can be further refined using HostPhinder [89] and PHISDetector [97], both useful as independent checks on PhaBOX CHERRY’s graph-based predictions. Overall, web-based workflows provide broad analytical coverage with minimal setup but remain constrained in scalability and flexibility compared to command-line approaches.

Command-line implementations enable scalable, high-sensitivity analysis, and integration of best-in-class tools across workflow stages (Fig. 2b). Assembly is performed using metaviralSPAdes [18] for short reads or viralFlye [34] for long reads, while Phables [32] improve recovery of complete genomes from fragmented metagenome assemblies. For identification, geNomad [7] is recommended as a primary tool due to its strong performance, but combining it with VirSorter2 [20] or VIBRANT [19] improves recall, reflecting the low concordance observed across identification tools [14]. CheckV [31] provides standardized genome quality assessment. Functional annotation follows a two-stage process: Pharokka [46] enables rapid gene calling and homology-based annotation, followed by Phold [8], which incorporates structure information to improve functional assignment. DRAM-v [48] can be incorporated to identify auxiliary metabolic gene detection where required. Taxonomic classification is performed using vConTACT3 [13] for hierarchical clustering yielding >95% ICTV agreement. VIRIDIC [63] can be applied for intergenomic similarity calculations in formal taxonomic assignments. Lifestyle prediction can be conducted using PhaTYP [73] or ProkBERT PhaStyle [75], both of which perform well on short contigs, while BACPHLIP [70] provides a simpler alternative for complete genomes. Defence system detection benefits from combining AntiDefenseFinder [77] and PADLOC [81, 82] with CRISPRCasTyper [80] providing detailed CRISPR subtype annotation. Host prediction is performed using iPHoP [98], which integrates multiple signal types to improve coverage. Given the consistently low agreement between tools across categories [14, 30], multi-tool strategies remain essential for robust phage genome analysis and tools should be selected based on input data type and required output regardless of user expertise. Furthermore, command-line implementations are stratified not merely by analytical intent but by computational requirements, that could range from laptop-scale to High-Performance Computing infrastructure.

Ultimately, the transition from fragmented, single-task execution to automated, end-to-end orchestration represents the most critical bottleneck in operationalizing these workflows. While the command-line ecosystem offers unparalleled analytical depth, it introduces substantial friction regarding data interoperability; the heterogeneous output formats of identification algorithms (e.g. geNomad) must be programmatically parsed and standardized to serve as inputs for downstream structural annotation or host prediction tools. To mitigate this fragility and ensure strict computational reproducibility, researchers could embed these individual tools within robust, containerized workflow management systems (such as Nextflow [104] and Snakemake [105]). Looking forward, the integration of agentic artificial intelligence (AI) frameworks capable of autonomous workflow orchestration [106], which translate biological queries into executable multi-tool pipelines, holds the potential to seamlessly bridge the usability gap, bringing the accessibility of web platforms to the rigorous environment of high-performance computing.

## Benchmarking, current limitations, and future directions

Across tool categories, successive computational paradigms have shifted performance baselines. In phage identification, geNomad increased reported MCCs from 0.813 for VirSorter2 to 0.953 [7]. In functional annotation, Phold’s structure-informed approach assigns functions to >50% of genes in a typical phage genome, compared with ~35% for homology-only workflows [8]. Lifestyle prediction has moved from complete-genome classifiers such as PHACTS to fragment-compatible foundation-model approaches, with ProkBERT PhaStyle reporting balanced accuracies of 0.88–0.93 on 500 bp fragments [75]. Host prediction has similarly benefited from ensemble learning, with iPHoP increasing host predictions rates by up to 13-fold at controlled false discovery rate [98], while vConTACT3 achieves greater than 95% agreement with ICTV taxonomy across multiple ranks [13].

Despite these gains, performance remains strongly dependent on input length, dataset composition, and evaluation design. Short contigs, particularly those below 3 kb, remain problematic across categories. DeepVirFinder’s area under the ROC curve (AUROC) decreases from ~0.98 at 3 kb to 0.93 at 300 bp [107], and DeePhage accuracy drops from 88.9% on 1200–1800 bp fragments to 76.2% on 100–400 bp fragments. Host prediction benchmarks rarely report length-stratified performance, although tools such as WIsH show reduced genus-level accuracy as candidate-host space expands. As short contigs dominate many metagenomic assemblies, this length dependence directly limits sensitivity and specificity.

Independent benchmarks further show that tool performance is highly dataset specific. Ho *et al*. found that only 89.7% of true phage contigs were detected by more than half of the tools tested, but only 11.6% were detected by all 10 tools tested [14]. Wu *et al*. reported true positive rates ranging from 0% to 97% across tools and datasets from different biomes [30]. These results indicate that individual tools capture partially overlapping subsets of viral diversity rather than a common, stable signal. Multi-tool workflows are therefore currently more robust than any single classifier, though they increase computational burden and complicate interpretation when predictions disagree [108]. Supplementary Table 1 separates metrics reported by tool developers from those obtained in independent evaluations and records, for each, the dataset and main caveats. Only the independent benchmarks conducted by Ho *et al*., Wu *et al*., and Shang *et al*. assess multiple tools on shared data; developer-reported figures are best read as indicative of broad trends rather than exact tool-versus-tool rankings.

Three recurring data limitations explain much of this variability. First, supervised methods remain constrained by small datasets. For instance, lifestyle prediction still relies heavily on benchmark sets containing fewer than 2000 experimentally labelled phages [73]. Second, reference databases are taxonomically and ecologically biased, with cultured phages dominated by a small number of bacterial host genera [2]. Thirdly, random-test splits can inflate performance when closely related genomes appear in both training and test sets. Further benchmarks should therefore use phylogenetically separated test sets, report performance by contig length and novelty of data inputs, and evaluate tools on independent environmental and clinical datasets [108].

Consequently, exceptionally high-performance metrics for recent foundation models must be interpreted cautiously. With 75% of sequenced phages originating from just 30 bacterial genera, these metrics likely reflect algorithmic overfitting to a hyper-characterized sequence space rather than true generalizability. Until evaluated against phylogenetically isolated holdout sets, these high accuracy ceilings merely represent the limits of deeply biased reference databases.

Viral dark matter remains a major unresolved challenge. Homology-based annotation leaves a large fraction of phage proteins uncharacterized [39, 109]. Although structure-informed approaches such as Phold have improved annotation rates, roughly half of genes still lack confident functional assignments [8]. Furthermore, binary classification schemes (such as virulent versus temperate, or host versus nonhost) oversimplify biological continua, omitting information such as pseudolysogeny, chronic infection, broad-host-range infection, and phage-plasmid lifestyles. As deep learning and foundation-model tools become more accurate, their reduced interpretability will need to be balanced against biologically grounded features such as domains, genomic neighbourhoods, structural similarity, and experimentally validated receptors [109].

Foundation models partially address the labelled-data bottleneck by learning representations from large unlabelled sequence and protein corpora [75]. Protein language models such as ESM-2 now support host prediction, protein family classification, and defence-system discovery [110, 111], while structure-aware pipelines combining predicted protein structures, structural alphabets [103], and Foldseek enable functional inference beyond detectable sequence homology [8, 46]. These dependencies not only create powerful shared infrastructure but also ecosystem-level fragility: changes to core components such as gene callers, HMM databases, protein language models, or structural search tools can propagate through many downstream pipelines (Table 2). Reducing this fragility requires treating the upstream dependencies mapped in Table 2 as versioned, auditable infrastructure rather than fixed background utilities. Version-pinning and containerizing these shared components guards downstream pipelines against silent breakage when an upstream release alters model weights, default parameters, or database composition. Transparent model cards and explicit database version stamps make the provenance of each prediction traceable and its inputs reproducible and versioned benchmark datasets let the field detect performance regressions as soon as a core component is updated, rather than discovering them piecemeal, one tool at atime.

The field urgently needs a community benchmarking framework analogous to Critical Assessment of Structure Prediction (CASP) or Critical Assessment of Metagenome Interpretation (CAMI) [112, 113]. Such a benchmark should include phylogenetically separated train–test splits, standardized metrics, common reference datasets, contig-length strata, biome-specific test sets, negative controls, and clinically relevant tasks such as strain-level host prediction. Reporting should include not only accuracy, AUROC, F1 score, and MCC but also calibration, false discovery rate, runtime, memory usage, and failure modes [108, 114]. Without this standardization, apparent performance gains will remain difficult to compare across studies.

Clinical translation imposes the most stringent requirements. Phage therapy would benefit greatly from strain-level and ideally receptor-level host prediction, yet most current tools operate at genus or species level. Recent studies in *Escherichia* and *Klebsiella* show that strain-level prediction is feasible in restricted settings, but no broadly generalizable predictor exists across major clinical pathogens [103]. Closing this gap will require larger experimentally validated phage–host interaction datasets, receptor-binding protein annotation, bacterial receptor modelling, and transfer-learning strategies that generalize across genera.

Overall, the next phase of phage bioinformatics should depend less on producing additional standalone tools and more on rigorous evaluation, interoperable infrastructure, and clinically meaningful prediction targets [30]. Structure-informed annotation, foundation-model representations, and generative genome models are already expanding what can be inferred from phage sequences [8, 56, 57]. Their impact will depend on whether the field can pair these advances with standardized benchmarks, experimentally grounded validation, and methods that resolve phage–host specificity at the strain and receptor levels.

## Conclusions

Phage bioinformatics has moved through three computational paradigms in under a decade. Homology-based tools established the foundations of phage detection and annotation but remained bounded by reference database completeness. Machine learning classifiers bypassed that ceiling by learning statistical patterns from sequence composition, extending identification and classification to unculturable phages, yet introduced a new bottleneck in labelled training data. Foundation models pretrained on massive unlabelled corpora have now begun to lift both constraints simultaneously, enabling reference-independent classification, structure-informed functional annotation that narrows the viral dark matter gap, and the first generative models capable of producing viable synthetic phage genomes [115]. The field is therefore crossing a fundamental threshold: from computational analysis of natural phages to computational design of newones.

Several substantive gaps persist. Roughly half of phage genes still resist functional annotation even with structural methods; no generalizable strain-level host predictor exists for phage therapy; and binary classification frameworks of phage lifecycles oversimplify biological phenomena. The most urgent community need, however, is standardized community benchmarking. Tool creation is outpacing evaluation, leaving researchers without a reliable basis for tool selection. These open problems define the next phase of phage bioinformatics and form the agenda for the tool developers this review is intended to equip.

**Key Points**

- Over 80 bioinformatics tools across eight functional categories now support phage research. The majority were published between 2020 and 2025, reflecting a rapid expansion driven by foundation-model architectures and the increasing clinical demand for phage therapy.
- Three computational paradigms (sequence homology, machine learning, and foundation models) have continually raised performance ceilings. Notably, foundation-model architectures now achieve Matthews correlation coefficients in identification tasks above 0.95 and have raised functional annotation from roughly 35% to over 50% of phage genes via structure-informed prediction.
- A small set of foundation components (gene callers, homology search engines, protein language models, and structural search tools) underpins end-user phage tools. While this establishes a powerful enabling infrastructure, it simultaneously introduces ecosystem-level fragility that is highly susceptible to upstream dependency shifts.
- Scalable strain-level host prediction remains unattainable. Current tools operate at the genus or species level, whereas phage therapy requires receptor-level specificity. Bridging this resolution gap represents one of the field’s most consequential unmet clinical needs.
- Methodological development is currently outpacing rigorous evaluation, with true-positive rates fluctuating substantially between 0% and 97% across disparate datasets. The community urgently needs a standardized benchmarking consortium, analogous to CASP or CAMI, using phylogenetically separated test sets to ensure robust, reproducible validation.

## Supplementary Material

Supplementary_material_bbag494

## Data Availability

No new data were generated or analysed in support of this review. All tools and databases discussed are publicly available and referenced in thetext.

## References

[1] Camargo AP, Nayfach S, Chen I-MA et al. IMG/VR v4: an expanded database of uncultivated virus genomes within a framework of extensive functional, taxonomic, and ecological metadata. *Nucleic Acids Res* 2023;51:D733–43. 10.1093/nar/gkac1037. 36399502 PMC9825611

[2] Cook R, Brown N, Redgwell T et al. INfrastructure for a PHAge REference database: identification of large-scale biases in the current collection of cultured phage genomes. *PHAGE Ther Appl Res* 2021;2:214–23. 10.1089/phage.2021.0007 PMC9041510 36159887

[3] Roux S, Enault F, Hurwitz BL et al. VirSorter: mining viral signal from microbial genomic data. *PeerJ* 2015;3:e985. 10.7717/peerj.985. 26038737 PMC4451026

[4] Bin Jang H, Bolduc B, Zablocki O et al. Taxonomic assignment of uncultivated prokaryotic virus genomes is enabled by gene-sharing networks. *Nat Biotechnol* 2019;37:632–9. 10.1038/s41587-019-0100-8. 31061483

[5] Ren J, Ahlgren NA, Lu YY et al. VirFinder: a novel k-mer based tool for identifying viral sequences from assembled metagenomic data. *Microbiome* 2017;5:69. 10.1186/s40168-017-0283-5. 28683828 PMC5501583

[6] Shang J, Jiang J, Sun Y . Bacteriophage classification for assembled contigs using graph convolutional network. *Bioinformatics* https://github.com/KennthShang/PhaGCN 2021;37:i25–33. 10.1093/bioinformatics/btab293. PMC8275337 34252923

[7] Camargo AP et al. Identification of mobile genetic elements with geNomad. *Nat Biotechnol* 2023;42:1303–12. 37735266 10.1038/s41587-023-01953-y PMC11324519

[8] Bouras G, Grigson SR, Mirdita M et al. Protein structure-informed bacteriophage genome annotation with Phold. *Nucleic Acids Res* 2026;54:gkaf1448. 10.1093/nar/gkaf1448. PMC12774648 41495893

[9] Wendling CC, Vasse M, Wielgoss S. Phage quest: a beginner’s guide to explore viral diversity in the prokaryotic world. *Brief Bioinform* 2025;26:bbaf449. 10.1093/bib/bbaf449. 40900113 PMC12406692

[10] Brister JR, Ako-adjei D, Bao Y et al. NCBI viral genomes resource. *Nucleic Acids Res* 2014;43:D571–7. 10.1093/nar/gku1207. 25428358 PMC4383986

[11] Russell DA, Hatfull GF. PhagesDB: the actinobacteriophage database. *Bioinformatics* 2016;33:784–6. 10.1093/bioinformatics/btw711. PMC5860397 28365761

[12] ICTV. ICTV Virus Metadata Resource (VMR). 2026. 10.5281/zenodo.18808244.

[13] Bolduc B, Zablocki O, Turner D et al. Machine learning enables scalable and systematic hierarchical virus taxonomy. *Nat Biotechnol* 2025. 10.1038/s41587-025-02946-9 (advance online publication). 41420049

[14] Ho SFS, Wheeler NE, Millard AD et al. Gauge your phage: benchmarking of bacteriophage identification tools in metagenomic sequencing data. *Microbiome* 2023;11:84. 10.1186/s40168-023-01533-x. 37085924 PMC10120246

[15] Amgarten D, Braga LPP, da Silva AM et al. MARVEL, a tool for prediction of bacteriophage sequences in metagenomic bins. *Front Genet* 2018;9:304. 10.3389/fgene.2018.00304. PMC6090037 30131825

[16] Auslander N, Gussow AB, Benler S et al. Seeker: alignment-free identification of bacteriophage genomes by deep learning. *Nucleic Acids Res* 2020;48:e121. 10.1093/nar/gkaa856. 33045744 PMC7708075

[17] Fang Z, Tan J, Wu S et al. PPR-meta: a tool for identifying phages and plasmids from metagenomic fragments using deep learning. *GigaScience* 2019;8:giz066. 10.1093/gigascience/giz066. PMC6586199 31220250

[18] Antipov D, Raiko M, Lapidus A et al. Metaviral SPAdes: assembly of viruses from metagenomic data. *Bioinformatics* 2020;36:4126–9. 10.1093/bioinformatics/btaa490. 32413137

[19] Kieft K, Zhou Z, Anantharaman K. VIBRANT: automated recovery, annotation and curation of microbial viruses, and evaluation of viral community function from genomic sequences. *Microbiome* 2020;8:90. 10.1186/s40168-020-00867-0. 32522236 PMC7288430

[20] Guo J, Bolduc B, Zayed AA et al. VirSorter2: a multi-classifier, expert-guided approach to detect diverse DNA and RNA viruses. *Microbiome* 2021;9:37. 10.1186/s40168-020-00990-y. PMC7852108 33522966

[21] Jurtz VI, Villarroel J, Lund O et al. MetaPhinder—identifying bacteriophage sequences in metagenomic data sets. *PLoS One* 2016;11:e0163111. 10.1371/journal.pone.0163111. 27684958 PMC5042410

[22] Shang J, Tang X, Guo R et al. Accurate identification of bacteriophages from metagenomic data using transformer. *Brief Bioinform* 2022;23:bbac258. 10.1093/bib/bbac258. PMC9294416 35769000

[23] Bai Z, Zhang YZ, Miyano S et al. Identification of bacteriophage genome sequences with representation learning. *Bioinformatics* 2022;38:4264–70. 10.1093/bioinformatics/btac509. 35920769 PMC9477532

[24] Ji Y, Zhou Z, Liu H et al. DNABERT: pre-trained bidirectional encoder representations from transformers model for DNA-language in genome. *Bioinformatics* 2021;37:2112–20. 10.1093/bioinformatics/btab083. 33538820 PMC11025658

[25] Akhter S, Aziz RK, Edwards RA. PhiSpy: a novel algorithm for finding prophages in bacterial genomes that combines similarity- and composition-based strategies. *Nucleic Acids Res* 2012;40:e126–6. 10.1093/nar/gks406. 22584627 PMC3439882

[26] Arndt D, Grant JR, Marcu A et al. PHASTER: a better, faster version of the PHAST phage search tool. *Nucleic Acids Res* 2016;44:W16–21. 10.1093/nar/gkw387. 27141966 PMC4987931

[27] Wishart DS, Han S, Saha S et al. PHASTEST: faster than PHASTER, better than PHAST. *Nucleic Acids Res* 2023;51:W443–50. 10.1093/nar/gkad382. 37194694 PMC10320120

[28] Gauthier CH, Abad L, Venbakkam AK et al. DEPhT: a novel approach for efficient prophage discovery and precise extraction. *Nucleic Acids Res* 2022;50:e75–5. 10.1093/nar/gkac273. 35451479 PMC9303363

[29] Sirén K, Millard A, Petersen B et al. Rapid discovery of novel prophages using biological feature engineering and machine learning. *NAR Genomics Bioinform* 2021;3:lqaa109. 10.1093/nargab/lqaa109. PMC7787355 33575651

[30] Wu S, Fang Z, Tan J et al. Benchmarking computational tools for virus identification in metagenomes across biomes. *Microbiome* 2024;12:215.39438898

[31] Nayfach S, Camargo AP, Schulz F et al. CheckV assesses the quality and completeness of metagenome-assembled viral genomes. *Nat Biotechnol* 2020;39:578–85. 33349699 10.1038/s41587-020-00774-7 PMC8116208

[32] Mallawaarachchi V, Roach MJ, Decewicz P et al. Phables: from fragmented assemblies to high-quality bacteriophage genomes. *Bioinformatics* 2023;39:btad586. 10.1093/bioinformatics/btad586. PMC10563150 37738590

[33] Bankevich A, Nurk S, Antipov D et al. SPAdes: a new genome assembly algorithm and its applications to single-cell sequencing. *J Comput Biol* 2012;19:455–77. 10.1089/cmb.2012.0021. 22506599 PMC3342519

[34] Antipov D, Rayko M, Kolmogorov M et al. viralFlye: assembling viruses and identifying their hosts from long-read metagenomics data. *Genome Biol* 2022;23:57. 10.1186/s13059-021-02566-x. 35189932 PMC8862349

[35] Chen L, Banfield JF. COBRA improves the completeness and contiguity of viral genomes assembled from metagenomes. *Nat Microbiol* 2024;9:737–50. 10.1038/s41564-023-01598-2. 38321183 PMC10914622

[36] Kieft K, Adams A, Salamzade R et al. vRhyme enables binning of viral genomes from metagenomes. *Nucleic Acids Res* 2022;50:e83. 10.1093/nar/gkac341. 35544285 PMC9371927

[37] Zolfo M, Pinto F, Asnicar F et al. Detecting contamination in viromes using ViromeQC. *Nat Biotechnol* 2019;37:1408–12. 10.1038/s41587-019-0334-5. 31748692

[38] Gilchrist CLM, Chooi Y-H. Clinker & clustermap.Js: automatic generation of gene cluster comparison figures. *Bioinformatics* 2021;37:2473–5. 10.1093/bioinformatics/btab007. 33459763

[39] Hatfull GF, Hendrix RW. Bacteriophages and their genomes. *Curr Opin Virol* 2011;1:298–303. 10.1016/j.coviro.2011.06.009. 22034588 PMC3199584

[40] Hatfull GF . Dark matter of the biosphere: the amazing world of bacteriophage diversity. *J Virol* 2015;89:8107–10. 10.1128/JVI.01340-15. 26018169 PMC4524254

[41] McNair K, Zhou C, Dinsdale EA et al. PHANOTATE: a novel approach to gene identification in phage genomes. *Bioinformatics* 2019;35:4537–42. 10.1093/bioinformatics/btz265. 31329826 PMC6853651

[42] Hyatt D, Chen GL, LoCascio PF et al. Prodigal: prokaryotic gene recognition and translation initiation site identification. *BMC Bioinformatics* 2010;11:119. 10.1186/1471-2105-11-119. PMC2848648 20211023

[43] Besemer J, Lomsadze A, Borodovsky M. GeneMarkS: a self-training method for prediction of gene starts in microbial genomes. Implications for finding sequence motifs in regulatory regions. *Nucleic Acids Res* 2001;29:2607–18. 10.1093/nar/29.12.2607. 11410670 PMC55746

[44] Kelley DR, Liu B, Delcher AL et al. Gene prediction with glimmer for metagenomic sequences augmented by classification and clustering. *Nucleic Acids Res* 2012;40:e9. 10.1093/nar/gkr1067. 22102569 PMC3245904

[45] Terzian P, Olo Ndela E, Galiez C et al. PHROG: families of prokaryotic virus proteins clustered using remote homology. *NAR Genomics Bioinform* 2021;3:lqab067. 10.1093/nargab/lqab067. PMC8341000 34377978

[46] Bouras G, Nepal R, Houtak G et al. Pharokka: a fast scalable bacteriophage annotation tool. *Bioinformatics* 2023;39:btac776. 10.1093/bioinformatics/btac776. PMC9805569 36453861

[47] Ecale Zhou CL, Kimbrel J, Edwards R et al. MultiPhATE2: code for functional annotation and comparison of phage genomes. *G3 GenesGenomesGenetics* 2021;11:jkab074. 10.1093/g3journal/jkab074. PMC8104953 33734357

[48] Shaffer M, Borton MA, McGivern BB et al. DRAM for distilling microbial metabolism to automate the curation of microbiome function. *Nucleic Acids Res* 2020;48:8883–900. 10.1093/nar/gkaa621. 32766782 PMC7498326

[49] Zimmermann L, Stephens A, Nam SZ et al. A completely reimplemented MPI bioinformatics toolkit with a new HHpred server at its Core. *J Mol Biol* 2018;430:2237–43. 10.1016/j.jmb.2017.12.007. 29258817

[50] Grazziotin AL, Koonin EV, Kristensen DM. Prokaryotic virus orthologous groups (pVOGs): a resource for comparative genomics and protein family annotation. *Nucleic Acids Res* 2016;45:D491–8. 10.1093/nar/gkw975. 27789703 PMC5210652

[51] Cantu VA, Salamon P, Seguritan V et al. PhANNs, a fast and accurate tool and web server to classify phage structural proteins. *PLoS Comput Biol* 2020;16:e1007845. 10.1371/journal.pcbi.1007845. 33137102 PMC7660903

[52] Albin D, Ramsahoye M, Kochavi E et al. PhageScanner: a reconfigurable machine learning framework for bacteriophage genomic and metagenomic feature annotation. Front Microbiol 2024;15:1446097. 10.3389/fmicb.2024.1446097. PMC11442244 39355420

[53] Flamholz ZN, Biller SJ, Kelly L. Large language models improve annotation of prokaryotic viral proteins. *Nat Microbiol* 2024;9:537–49. 10.1038/s41564-023-01584-8. 38287147 PMC11311208

[54] Boulay A, Leprince A, Enault F et al. Empathi: embedding-based phage protein annotation tool by hierarchical assignment. *Nat Commun* 2025;16:9114. 10.1038/s41467-025-64177-5. 41087364 PMC12521583

[55] Guan J et al. GOPhage: protein function annotation for bacteriophages by integrating the genomic context. *Brief Bioinform* 2025;26:bbaf014. 10.1093/bib/bbaf014 PMC11751364 39838963

[56] Heinzinger M, Weissenow K, Sanchez JG et al. Bilingual language model for protein sequence and structure. *NAR Genomics Bioinform* 2024;6:lqae150. 10.1093/nargab/lqae150. PMC11616678 39633723

[57] van Kempen M, Kim SS, Tumescheit C et al. Fast and accurate protein structure search with Foldseek. *Nat Biotechnol* 2023;42:243–6. 37156916 10.1038/s41587-023-01773-0 PMC10869269

[58] Mishra PM, Verma NC, Rao C et al. Intrinsically disordered proteins of viruses: involvement in the mechanism of cell regulation and pathogenesis. *Prog Mol Biol Transl Sci* 2020;174:1–78. 32828463 10.1016/bs.pmbts.2020.03.001 PMC7129803

[59] Abramson J, Adler J, Dunger J et al. Accurate structure prediction of biomolecular interactions with AlphaFold 3. *Nature* 2024;630:493–500. 10.1038/s41586-024-07487-w. 38718835 PMC11168924

[60] Turner D, Shkoporov AN, Lood C et al. Abolishment of morphology-based taxa and change to binomial species names: 2022 taxonomy update of the ICTV bacterial viruses subcommittee. *Arch Virol* 2023;168:74. 10.1007/s00705-022-05694-2. 36683075 PMC9868039

[61] Meier-Kolthoff JP, Göker M. VICTOR: genome-based phylogeny and classification of prokaryotic viruses. *Bioinformatics* 2017;33:3396–404. 10.1093/bioinformatics/btx440. 29036289 PMC5860169

[62] Nishimura Y, Yoshida T, Kuronishi M et al. ViPTree: the viral proteomic tree server. *Bioinformatics* 2017;33:2379–80. 10.1093/bioinformatics/btx157. 28379287

[63] Moraru C, Varsani A, Kropinski AM. VIRIDIC—A novel tool to calculate the intergenomic similarities of prokaryote-infecting viruses. *Viruses* 2020;12:1268. 33172115 10.3390/v12111268 PMC7694805

[64] Millard A, Denise R, Lestido M et al. taxMyPhage: automated taxonomy of dsDNA phage genomes at the genus and species level. *PHAGE Ther Appl Res* 2025;6:5–11. 10.1089/phage.2024.0050 PMC12060842 40351403

[65] Mayne R, Aiewsakun P, Turner D et al. GRAViTy-V2: a grounded viral taxonomy application. *NAR Genomics Bioinform* 2024;6:lqae183. 10.1093/nargab/lqae183. PMC11655284 39703433

[66] Shang J, Jiang J, Sun Y. Bacteriophage classification for assembled contigs using graph convolutional network. *Bioinformatics* 2021;37:i25–33. 10.1093/bioinformatics/btab293. 34252923 PMC8275337

[67] Shang J, Peng C, Liao H et al. PhaBOX: a web server for identifying and characterizing phage contigs in metagenomic data. *Bioinform Adv* 2023;3:vbad101. 10.1093/bioadv/vbad101. PMC10460485 37641717

[68] Shang J, Peng C, Guan J et al. PhaBOX2: an enhanced web server for discovering and analyzing viral contigs in metagenomic data. *Nucleic Acids Res* 2026;54:W169–76. 10.1093/nar/gkag382. 42023515 PMC13355064

[69] Smug BJ, Szczepaniak K, Rocha EPC et al. Ongoing shuffling of protein fragments diversifies core viral functions linked to interactions with bacterial hosts. *Nat Commun* 2023;14:7460. 10.1038/s41467-023-43236-9. 38016962 PMC10684548

[70] Hockenberry AJ, Wilke CO. BACPHLIP: predicting bacteriophage lifestyle from conserved protein domains. *PeerJ* 2021;9:e11396. 10.7717/peerj.11396. 33996289 PMC8106911

[71] McNair K, Bailey BA, Edwards RA. PHACTS, a computational approach to classifying the lifestyle of phages. *Bioinformatics* 2012;28:614–8. 10.1093/bioinformatics/bts014. 22238260 PMC3289917

[72] Wu S, Fang Z, Tan J et al. DeePhage: distinguishing virulent and temperate phage-derived sequences in metavirome data with a deep learning approach. *GigaScience* 2021;10:giab056. 10.1093/gigascience/giab056. PMC8427542 34498685

[73] Shang J, Tang X, Sun Y. PhaTYP: predicting the lifestyle for bacteriophages using BERT. *Brief Bioinform* 2022;24:bbac487. 10.1093/bib/bbac487. PMC9851330 36659812

[74] Zhang Y, Mao M, Zhang R et al. DeepPL: a deep-learning-based tool for the prediction of bacteriophage lifecycle. *PLoS Comput Biol* 2024;20:e1012525. 10.1371/journal.pcbi.1012525. 39418300 PMC11521287

[75] Juhász J, Ligeti-Nagy N, Bodnár B et al. ProkBERT PhaStyle: accurate phage lifestyle prediction with pretrained genomic language models. *Bioinform Adv* 2024;5:vbaf188. 10.1093/bioadv/vbaf188. PMC12603353 41230491

[76] Zhou, Z., Yanrong Ji, Weijian Li, Pratik Dutta, Ramana Davuluri., et al. DNABERT-2: Efficient Foundation Model and Benchmark for Multi-Species Genome. in (2024). doi:10.48550/arXiv.2306.15006.

[77] Tesson F, Hervé A, Mordret E et al. Systematic and quantitative view of the antiviral arsenal of prokaryotes. *Nat Commun* 2022;13:2561. 10.1038/s41467-022-30269-9. 35538097 PMC9090908

[78] Biswas A, Staals RHJ, Morales SE et al. CRISPRDetect: a flexible algorithm to define CRISPR arrays. *BMC Genomics* 2016;17:356. 10.1186/s12864-016-2627-0. PMC4869251 27184979

[79] Couvin D, Bernheim A, Toffano-Nioche C et al. CRISPRCasFinder, an update of CRISRFinder, includes a portable version, enhanced performance and integrates search for Cas proteins. *Nucleic Acids Res* 2018;46:W246–51. 10.1093/nar/gky425. 29790974 PMC6030898

[80] Russel J, Pinilla-Redondo R, Mayo-Muñoz D et al. CRISPRCasTyper: automated identification, annotation, and classification of CRISPR-Cas loci. *CRISPR J* 2020;3:462–9. 10.1089/crispr.2020.0059. 33275853

[81] Payne LJ, Todeschini TC, Wu Y et al. Identification and classification of antiviral defence systems in bacteria and archaea with PADLOC reveals new system types. *Nucleic Acids Res* 2021;49:10868–78. 10.1093/nar/gkab883. 34606606 PMC8565338

[82] Payne LJ, Meaden S, Mestre MR et al. PADLOC: a web server for the identification of antiviral defence systems in microbial genomes. *Nucleic Acids Res* 2022;50:W541–50. 10.1093/nar/gkac400. 35639517 PMC9252829

[83] Millman A, Melamed S, Leavitt A et al. An expanded arsenal of immune systems that protect bacteria from phages. *Cell Host Microbe* 2022;30:1556–1569.e5. 10.1016/j.chom.2022.09.017. 36302390

[84] Yi H, Huang L, Yang B et al. AcrFinder: genome mining anti-CRISPR operons in prokaryotes and their viruses. *Nucleic Acids Res* 2020;48:W358–65. 10.1093/nar/gkaa351. 32402073 PMC7319584

[85] Wen Y, Zhang F, Jiang Y. AcaFinder: genome mining anti-CRISPR-associated genes. *mSystems* 2023;8:e00981–22. 10.1128/msystems.00817-22 PMC9765179 36413017

[86] Eitzinger S, Asif A, Watters KE et al. Machine learning predicts new anti-CRISPR proteins. *Nucleic Acids Res* 2020;48:4698–708. 10.1093/nar/gkaa219. 32286628 PMC7229843

[87] Li Y, Wei Y, Xu S et al. AcrNET: predicting anti-CRISPR with deep learning. *Bioinformatics* 2023;39:btad259. 10.1093/bioinformatics/btad259. PMC10174705 37084259

[88] DeWeirdt PC, Mahoney EM, Laub MT. DefensePredictor: a machine learning model to discover prokaryotic immune systems. Science 2026;392:eadv7924. 10.1126/science.adv7924. PMC13092281 41926577

[89] Villarroel J, Kleinheinz K, Jurtz V et al. HostPhinder: a phage host prediction tool. *Viruses* 2016;8:116. 10.3390/v8050116. 27153081 PMC4885074

[90] Galiez C, Siebert M, Enault F et al. WIsH: who is the host? Predicting prokaryotic hosts from metagenomic phage contigs. *Bioinformatics* 2017;33:3113–4. 10.1093/bioinformatics/btx383. 28957499 PMC5870724

[91] Zhang R, Mirdita M, Levy Karin E et al. SpacePHARER: sensitive identification of phages from CRISPR spacers in prokaryotic hosts. *Bioinformatics* 2021;37:3364–6. 10.1093/bioinformatics/btab222. 33792634 PMC8504623

[92] Coutinho FH, Zaragoza-Solas A, López-Pérez M et al. RaFAH: host prediction for viruses of bacteria and archaea based on protein content. *Patterns* 2021;2:100274. 10.1016/j.patter.2021.100274. 34286299 PMC8276007

[93] Shang J, Sun Y. CHERRY: a computational metHod for accuratE pRediction of virus–pRokarYotic interactions using a graph encoder–decoder model. *Brief Bioinform* 2022;23:bbac182. 10.1093/bib/bbac182. PMC9487644 35595715

[94] Shang J, Sun Y. Predicting the hosts of prokaryotic viruses using GCN-based semi-supervised learning. *BMC Biol* 2021;19:250. 10.1186/s12915-021-01180-4. 34819064 PMC8611875

[95] Amgarten D, Iha BKV, Piroupo CM et al. vHULK, a new tool for bacteriophage host prediction based on annotated genomic features and neural networks. *PHAGE* 2022;3:204–12. 10.1089/phage.2021.0016. 36793881 PMC9917316

[96] Zielezinski A, Deorowicz S, Gudyś A. PHIST: fast and accurate prediction of prokaryotic hosts from metagenomic viral sequences. *Bioinformatics* 2021;38:1447–9. 10.1093/bioinformatics/btab837. PMC8826084 34904625

[97] Zhou F, Gan R, Zhang F et al. PHISDetector: a tool to detect diverse In silico phage–host interaction signals for Virome studies. *Genomics Proteomics Bioinformatics* 2022;20:508–23. 10.1016/j.gpb.2022.02.003. 35272051 PMC9801046

[98] Roux S, Camargo AP, Coutinho FH et al. iPHoP: an integrated machine learning framework to maximize host prediction for metagenome-derived viruses of archaea and bacteria. *PLoS Biol* 2023;21:e3002083. 10.1371/journal.pbio.3002083. 37083735 PMC10155999

[99] Liu F, Zhao Z, Liu Y. PHPGAT: predicting phage hosts based on multimodal heterogeneous knowledge graph with graph attention network. *Brief Bioinform* 2024;26:bbaf017. 10.1093/bib/bbaf017. PMC11745545 39833104

[100] Gonzales MEM, Ureta JC, Shrestha AMS. PHIStruct: improving phage–host interaction prediction at low sequence similarity settings using structure-aware protein embeddings. *Bioinformatics* 2025;41:btaf016. 10.1093/bioinformatics/btaf016. PMC11783280 39804673

[101] Chen Q, Zhao Z, Li M et al. MoEPH: an adaptive fusion-based LLM for predicting phage-host interactions in health informatics. *Front Microbiol* 2025;16:1634705. 10.3389/fmicb.2025.1634705. PMC12488635 41048513

[102] Klein-Sousa V, Roa-Eguiara A, Kielkopf CS et al. RBPseg: toward a complete phage tail fiber structure atlas. *Sci Adv* 2025;11:eadv0870. 10.1126/sciadv.adv0870. 40479047 PMC12143355

[103] Boeckaerts D, Stock M, Ferriol-González C et al. Prediction of Klebsiella phage-host specificity at the strain level. *Nat Commun* 2024;15:4355. 10.1038/s41467-024-48675-6. 38778023 PMC11111740

[104] Di Tommaso P, Chatzou M, Floden EW et al. Nextflow enables reproducible computational workflows. *Nat Biotechnol* 2017;35:316–9. 10.1038/nbt.3820. 28398311

[105] Köster J, Rahmann S. Snakemake—a scalable bioinformatics workflow engine. *Bioinformatics* 2012;28:2520–2. 10.1093/bioinformatics/bts480. 22908215

[106] Dip SA, Mallick D, Acharjee Shuvo U et al. Large language model agents for biological intelligence across genomics, proteomics, spatial biology, and biomedicine. *Brief Bioinform* 2026;27:bbag110. 10.1093/bib/bbag110. 41883029 PMC13017847

[107] Ren J, Song K, Deng C et al. Identifying viruses from metagenomic data using deep learning. *Quant Biol* 2020;8:64–77. 10.1007/s40484-019-0187-4. 34084563 PMC8172088

[108] Shang J, Peng C, Guan J et al. From genomic signals to prediction tools: a critical feature analysis and rigorous benchmark for phage–host prediction. *Brief Bioinform* 2025;26:bbaf626. 10.1093/bib/bbaf626. PMC12641615 41283812

[109] Grigson SR, Bouras G, Dutilh BE et al. Computational function prediction of bacteria and phage proteins. *Microbiol Mol Biol Rev* 2025;89:e0002225. 10.1128/mmbr.00022-25. 40824055 PMC12462290

[110] Lin Z, Akin H, Rao R et al. Evolutionary-scale prediction of atomic-level protein structure with a language model. *Science* 2023;379:1123–30. 10.1126/science.ade2574. 36927031

[111] Hayes T, Rao R, Akin H et al. Simulating 500 million years of evolution with a language model. *Science* 2025;387:850–8. 10.1126/science.ads0018. 39818825

[112] Kryshtafovych A, Schwede T, Topf M et al. Critical assessment of methods of protein structure prediction (CASP)—round XIV. *Proteins Struct Funct Bioinf* 2021;89:1607–17. 10.1002/prot.26237. PMC8726744 34533838

[113] Meyer F, Fritz A, Deng ZL et al. Critical assessment of metagenome interpretation: the second round of challenges. *Nat Methods* 2022;19:429–40. 10.1038/s41592-022-01431-4. 35396482 PMC9007738

[114] Gaborieau B, Vaysset H, Tesson F et al. Prediction of strain level phage–host interactions across the Escherichia genus using only genomic information. *Nat Microbiol* 2024;9:2847–61. 10.1038/s41564-024-01832-5. 39482383

[115] King SH, Driscoll CL, Li DB et al. Generative design of novel bacteriophages with genome language models. 2025. 10.1101/2025.09.12.675911. 42561074

