# Supplementary material for: Phage bioinformatics tools: a review of computational approaches for bacteriophage research
Source: Brief Bioinform. 2026 Sep 15;27(5):bbag494. doi: 10.1093/bib/bbag494 (PMC13577670; doi:10.1093/bib/bbag494)
Supplement: Supplementary file 1 [file supplementary_material_bbag494.zip › Suplementary Table 1.docx]

**Supplementary Table 1. Reported performance metrics: developer versus independent benchmarks.**

| **Task** | **Tool** | **Reported metric** | **Dataset / benchmark** | **Source** | **Key caveats** |
| --- | --- | --- | --- | --- | --- |
| Identification | VirSorter | >95% recall, 100% precision | contigs ≥10 kb | **Original** | recall collapses on <10 kb / novel lineages |
| Identification | VirFinder | 78× higher TPR than VirSorter at 1 kb (equal FPR) | own k-mer benchmark | **Original** | fold-change vs one tool; 2.4× at 3 kb, 1.8× at 5 kb |
| Identification | DeepVirFinder | AUROC 0.93 / 0.95 / 0.97 / 0.98 | 300 / 500 / 1000 / 3000 bp | **Original** | length-stratified; AUROC not comparable to MCC/F1 |
| Identification | geNomad | MCC 0.953, F1 0.973 (VirSorter2 MCC 0.813) | own IMG/VR-derived test | **Original** | shared-source train/test may inflate |
| Identification | PhaMer | +27% F1 over prior tools | mock community (real reads) | **Original** | relative improvement vs other tools |
| Identification | Kraken2 | precision 0.96 | mock community (Ho et al.) | **Independent** | high precision / low recall |
| Identification | cross-tool studies | 89.7% of contigs by >½ tools; 11.6% (775/6665) by all 10 | Ho et al. common set | **Independent** | shows low inter-tool concordance |
| Identification | cross-biome studies | true-positive rate 0–97% (FPR 0–30%) | Wu et al.: 9 tools × 8 datasets × 3 biomes | **Independent** | strong dataset / biome dependence |
| Prophage | PhiSpy | 94% of prophages detected (6% false-negative) | 50 bacterial genomes | **Original** | recall, not overall accuracy |
| Annotation | PhANNs | weighted F1 0.875 | 10 structural classes | **Original** | structural-protein classification only |
| Annotation | VPF-PLM | +29.4% annotation (26,770 new families) | EFAM ocean virome | **Original** | coverage gain, not correctness |
| Annotation | Empathi | annotation rate 16% → 33% | own (PHROGs) | **Original** | rate vs homology baseline |
| Annotation | Phold | >50% of genes vs ~35% homology-only | own | **Original** | coverage, not correctness |
| Taxonomy | vConTACT2 | 96% correspondence to genus-level ICTV | own | **Original** | genus only; leaves divergent outliers |
| Taxonomy | PhaGCN2 | recall 89.30%, precision 83.91% | ICTV2021 newly-added set (2,636) | **Original** | trained on ICTV2020; dataset-specific |
| Taxonomy | vConTACT3 | ICTV agreement 97.6% genus / 98.7% subfamily / 100% family & order | 4 viral realms | **Original** | agreement with ICTV labels, not novel-taxon discovery |
| Lifestyle | PHACTS | 99% precision, 88% sensitivity | 199 confident / 227 labelled | **Original** | complete genomes; degrades on partial |
| Lifestyle | BACPHLIP | 98.3% accuracy (415/423) vs 79% (PHACTS) | 423 test phages | **Original** | complete genomes only; not fragments |
| Lifestyle | DeePhage | 76.2% (100–400 bp) → 88.9% (1200–1800 bp) | own | **Original** | strongly length-dependent |
| Lifestyle | PhaTYP | 0.89 accuracy (100–400 bp) | ~1,867-phage benchmark | **Original** | small labelled set |
| Lifestyle | DeepPL | 94.65% accuracy | own | **Original** | DNABERT fine-tune |
| Lifestyle | ProkBERT PhaStyle | balanced acc 0.88–0.93, MCC 0.75–0.86 (500 bp) | own | **Original** | same small benchmark |
| Host | SpacePHARER | 1.4–4× more sensitive than BLASTN | own | **Original** | CRISPR-spacer signal only |
| Host | PHIST | +3 percentage points species-level vs alignment; ~2 orders faster | own | **Original** | speed / accuracy trade-off |
| Host | iPHoP | 1.5–13× more host predictions (controlled FDR) | own | **Original** | coverage gain; >300 GB DB |
| Host | PHIStruct | +5–6% F1 over BLASTp where train-test similarity <40% | own | **Original** | low-similarity regime |
| Host | PhageHostLearn | ROC AUC 81.8% in silico (79.3% in vitro) | Klebsiella | **Original** | single genus; strain level |
| Host | critical benchmark | feature analysis: train-test leakage inflates metrics | Shang et al. | **Independent** | basis for cautious reading of all host metrics |
