# Supplementary material for: Phage bioinformatics tools: a review of computational approaches for bacteriophage research
Source: Brief Bioinform. 2026 Sep 15;27(5):bbag494. doi: 10.1093/bib/bbag494 (PMC13577670; doi:10.1093/bib/bbag494)
Supplement: Supplementary file 1 [file supplementary_material_bbag494.zip › Supplementary Table 2.docx]

**Supplementary Table 2. Host-prediction tools grouped by the host resolution they achieve.**

| **Resolution** | **Tool** | **Approach / primary signal** | **Best-suited application** | **Key caveat** |
| --- | --- | --- | --- | --- |
| **Genus** | **WIsH** | Per-host Markov models (order 8) on candidate host genomes | Fast metagenomic host-range screening (ecological) | Genus level only; accuracy falls on short contigs (~28% at 1 kb) and is unreliable for phages with >4 tRNAs |
| **Genus** | **RaFAH** | Random Forest on bit-scores to 43,644 protein-cluster HMM profiles | Bacteria/archaea host inference from protein content (ecological) | Built for genus (species left to future work); better suited to genomes >=50% complete |
| **Genus** | **HostG** | GCN semi-supervised learning over virus-virus and virus-host similarity graph | Genus inference including previously unseen taxa (ecological) | Deliberately stops at genus for reliability; longer runtime than peers |
| **Genus** | **iPHoP** | Random-forest ensemble of RaFAH plus host-based tools (WIsH, VirHostMatcher, PHP) | Reliable genus calls at <10% FDR for metagenome-derived viruses | Genus by design - species/strain explicitly out of scope; >300 GB database; ~12 min per 5 genomes |
| **Genus** | **PHIStruct** | SaProt (650M) structure-aware embeddings of RBPs into an MLP | Genus calls among ESKAPEE pathogens at low sequence similarity | Resolves only to genus; needs RBP identification + predicted structures; +7-9% F1 vs ML and +5-6% vs BLASTp at <40% similarity |
| **Species** | **HostPhinder** | k-mer co-occurrence vs a reference database of 2,196 known-host phages | Species/genus calls for cultured-phage-like queries (ecological) | Reference-database bound (genus 81% / species 74%); weaker on novel phages |
| **Species** | **vHULK** | Neural network on alignment scores to pVOG protein families | Species (and genus) calls from annotated genomic features (ecological) | Small host repertoire (118 species / 77 genera); no confidence score at species level |
| **Species** | **CHERRY** | GCN encoder-decoder link prediction over a multimodal graph | Species calls; also reverse (which phages infect a given host) | Species accuracy ~78%; explicitly not optimised for strain level |
| **Species** | **PHIST** | Exact shared-k-mer counts between viral and candidate-host genomes | Fast, laptop-scale species-level screening (ecological) | Modest absolute accuracy (~29.5% species); ~2 orders of magnitude faster; robust to short contigs |
| **Species** | **PHISDetector** | Random-forest over 7 in-silico interaction signals (CRISPR, prophage, homology, composition) | Multi-signal species/genus calls for virome studies | Species 0.51 / genus 0.73 accuracy; ~30% of known interactions show no detectable signal |
| **Species** | **PHPGAT** | GATv2 graph attention over a multimodal phage-host knowledge graph | Species calls integrating RBP, CRISPR and 16S features | Small training set (1,040 pairs); degrades on genomes >150 kb; compute-heavy |
| **Species** | **SpacePHARER** | Protein-level CRISPR-spacer matching (MMseqs2, six-frame) | High-confidence hits where host CRISPR spacers exist | CRISPR signal only; resolves to a higher rank when the host is absent from the database |
| **Strain** | **PhageHostLearn** | XGBoost on ESM-2 embeddings of phage RBPs + bacterial K-locus receptors | Strain-level matching for therapeutic phage selection | Demonstrated only for Klebsiella; overfits K-locus type; ROC AUC 81.8% in silico / 79.3% in vitro |
| **Strain** | **MoEPH** | Mixture-of-Experts fusion of ProtBERT + ProT5 embeddings with statistical descriptors | Strain-level phage-host interaction scoring | Tiny private datasets (38-101 strains); 99.6% only on balanced data; interpretability-oriented |
| **Receptor / RBP** | **RBPseg** | ESMFold + sDp domain segmentation + AF2M modelling of tail-fibre RBPs | RBP structure atlas to inform specificity and phage engineering (therapeutic) | Characterises RBP structure, not host taxa; validated by cryo-EM on 5 fibres from 3 phages |
