# Supplementary material for: Phage bioinformatics tools: a review of computational approaches for bacteriophage research
Source: Brief Bioinform. 2026 Sep 15;27(5):bbag494. doi: 10.1093/bib/bbag494 (PMC13577670; doi:10.1093/bib/bbag494)
Supplement: Supplementary file 1 [file supplementary_material_bbag494.zip › Supplementary_Table_3.docx]

**Supplementary Table 3. Computational resources for the tools recommended in the web-based (Figure 2a) and command-line (Figure 2b) workflows.**

| **Workflow step (Fig. 2)** | **Tool** | **Interface** | **Compute tier** | **Resources required (as reported)** | **Key resource caveat** |
| --- | --- | --- | --- | --- | --- |
| **1. Identification** | PhaMer (PhaBOX) | Web (Fig. 2a) | Browser; GPU (local) | Runs in the PhaBOX web server; local version is a Transformer model | Web upload limits; local model GPU-suited |
|  | geNomad | CLI (Fig. 2b) | Laptop–Server (CPU) | “Computationally efficient and scalable”; marker-protein database + models downloaded once | Mixed output formats need parsing |
|  | VirSorter2 | CLI (Fig. 2b) | Server (CPU; thread-scalable) | ~28.6 h for 100,000 sequences (4.2 Gb) on 32 threads; memory nearly constant | CPU time dominated by the large viral HMM database |
|  | VIBRANT | CLI (Fig. 2b) | Laptop–Server (CPU) | HMM search plus neural-network (v-score) scoring | HMM-database-bound |
| **2. Assembly & QC** | SPAdes (Galaxy/KBase) | Web (Fig. 2a) | Browser; Server (local) | Assembly graph; memory scales with dataset | Hosted compute quotas |
|  | metaviralSPAdes | CLI (Fig. 2b) | Server (RAM) | Short-read (Illumina) viral assembler; RAM scales with metagenome | Complete genomes hard from short reads |
|  | viralFlye | CLI (Fig. 2b) | Server (RAM) | Long-read assembler (ONT / PacBio CLR / HiFi) | Requires long-read data |
|  | Phables | CLI (Fig. 2b) | Laptop (CPU) | Core methods run in <2 min with <4 GB memory | Needs an assembly graph as input |
|  | CheckV | Web + CLI | Laptop–Server (CPU) | Reference database of 15,958 profile HMMs (download) | Database download required |
| **3. Annotation** | PHROGs | Web (Fig. 2a) | Browser | Hosted profile-HMM database (used via HHpred / web) | Web query limits |
|  | HHpred | Web (Fig. 2a) | Browser | Hosted remote-homology (HMM–HMM) web server | Interactive; small batches |
|  | PHASTEST | Web (Fig. 2a) | Browser | Hosted prophage-annotation web server | Web upload limits |
|  | Pharokka | CLI (Fig. 2b) | Laptop (CPU) | <5 min for an average 50-kb bacteriophage on one CPU | Homology-based; hypothetical genes remain |
|  | Phold | CLI (Fig. 2b) | GPU | ProstT5 structure step ~66 min for 1,419 genomes on an NVIDIA A100 | GPU strongly recommended |
|  | DRAM-v | CLI (Fig. 2b) | Server (CPU; DB) | HMM/database annotation of AMGs; needs reference databases | Large database downloads |
| **4. Taxonomy** | PhaGCN (PhaBOX) | Web (Fig. 2a) | Browser; GPU (local) | PhaBOX web module; local version is a graph CNN | Web upload limits |
|  | VICTOR | Web (Fig. 2a) | Browser | Hosted genome-distance phylogeny web server | Limited genomes per run |
|  | ViPTree | Web (Fig. 2a) | Browser | Hosted proteomic-tree web server | Limited genomes per run |
|  | vConTACT3 | CLI (Fig. 2b) | Server (CPU/RAM) | Gene-sharing network; scales to large sets | Divergent phages fall into unassigned clusters |
|  | VIRIDIC | CLI (Fig. 2b) | Laptop (CPU) | All-vs-all pairwise identity; small sets | Scales quadratically with genome count |
| **5. Lifestyle** | PhaTYP (PhaBOX) | Web + CLI | Browser; Laptop–GPU (local) | BERT model (trained on 4× GTX 2080 Ti, 24 GB); inference via PhaBOX | Fragment-capable |
|  | ProkBERT PhaStyle | CLI (Fig. 2b) | Laptop–GPU | Genomic language model with GPU support (A100 in evaluation); CPU-capable | Fragment-capable |
|  | BACPHLIP | CLI (Fig. 2b) | Laptop (CPU) | HMM plus random forest; complete genomes only | Not for fragments |
| **6. Defence** | DefenseFinder | Web + CLI | Laptop (CPU) | HMM homologue search against a curated system database | Catalogue-bound (known systems only) |
|  | AntiDefenseFinder | CLI (Fig. 2b) | Laptop (CPU) | DefenseFinder suite; HMM homologue search | Catalogue-bound |
|  | PADLOC | Web + CLI | Laptop (CPU) | HMM-based homologue searches against precomputed databases | Catalogue-bound |
|  | CRISPRCasFinder | Web + CLI | Laptop (CPU) | Rule- and HMM-based CRISPR/Cas detection | — |
|  | CRISPRCasTyper | CLI (Fig. 2b) | Laptop (CPU) | ML/rule-based CRISPR subtype typing | — |
|  | AcrNET | CLI (Fig. 2b) | GPU (deep learning) | Deep-learning (ESM-based) anti-CRISPR predictor | Hosted web server capped input at 3 short proteins after RAM reached 49 GB at concurrency 5; run locally for larger jobs |
|  | AcaFinder | CLI (Fig. 2b) | Laptop (CPU) | HMM/homology plus guilt-by-association | Catalogue-bound |
| **7. Host prediction** | CHERRY (PhaBOX) | Web (Fig. 2a) | Browser; GPU (local) | PhaBOX web module; local version is a graph CNN | Mainly genus-level |
|  | HostPhinder | Web + CLI | Laptop (CPU) | k-mer similarity to reference phage genomes | Reference-bound |
|  | PHISDetector | Web + CLI | Server (CPU) | Multi-signal (CRISPR, homology, oligonucleotide) detection | Several analyses per run |
|  | iPHoP | CLI (Fig. 2b) | HPC / large-DB | Reference database >300 GB; ~12 min for 5 complete genomes | Storage is the main barrier; genus-level |
|  | PHIStruct | CLI (Fig. 2b) | GPU | Structure-aware protein language model (SaProt) embeddings of RBPs | GPU-suited; for low-similarity hosts |
